# Association between gestational age-specific weight gain in pregnancy and risk of adverse perinatal outcomes: a secondary analysis of the INTERBIO-21st Fetal Study

**DOI:** 10.1016/j.ajcnut.2025.04.012

**Published:** 2025-04-16

**Authors:** Nusrat Jabin, Lucas Malla, Grieven Otieno, Leila Cheikh Ismail, INTERBIO-21st Consortium, Fernando C Barros, James Berkley, Maria Carvalho, Shama Munim, Shane Norris, Francois Nosten, Aris T Papageorghiou, Stephen H Kennedy, Jose Villar, Eric O Ohuma

**Affiliations:** 6 Programa de Pós-Graduação em Saúde e Comportamento, Universidade Federal de Pelotas, Pelotas, RS, Brazil; 7 KEMRI-Coast Centre for Geographical Medicine and Research, University of Oxford, Kilifi, Kenya; 8 Centre for Tropical Medicine and Global Health, University of Oxford, Oxford, UK; 9 Faculty of Health Sciences, Aga Khan University, Nairobi, Kenya; 10 Department of Obstetrics and Gynaecology, Division of Women and Child Health, Aga Khan University, Karachi, Pakistan; 11 SAMRC Developmental Pathways For Health Research Unit, Department of Paediatrics & Child Health, University of the Witwatersrand, Johannesburg, South Africa; 12 Shoklo Malaria Research Unit, Mahidol-Oxford Tropical Medicine Research Unit, Faculty of Tropical Medicine, Mahidol University, Mae Sot, Thailand; 1 Centre for Tropical Medicine and Global Health, University of Oxford, Oxford, United Kingdom; 2 Kenya Paediatric Research Consortium, Nairobi, Kenya; 3 Faculty of Epidemiology and Population Health, London School of Hygiene & Tropical Medicine, London, United Kingdom; 4 Nuffield Department of Women’s & Reproductive Health, University of Oxford, Oxford, United Kingdom; 5 College of Health Science, University of Sharjah, Sharjah, UAE

**Keywords:** gestational weight gain, gestational age, adverse maternal and neonatal adverse outcomes, INTERGROWTH-21st, INTERBIO-21st, Institute of Medicine

## Abstract

**Background:**

Gestational weight gain (GWG) is a potentially modifiable factor that can influence perinatal health outcomes.

**Objectives:**

This study aims to investigate the association between gestational age (GA)-specific weight gain and adverse perinatal outcomes.

**Methods:**

This study is a secondary analysis of the INTERBIO-21st Fetal Study, a prospective, longitudinal cohort conducted from 8 February, 2012 to 30 November, 2019, across 6 sites in Brazil, Kenya, Pakistan, South Africa, Thailand, and the United Kingdom. A total of 3354 pregnant females, aged ≥18 y with a body mass index (BMI) <35 kg/m^2^, initiated antenatal care before 14 wk of gestation. Weight was measured at 5 ± 1 wk intervals from 14 to 40 wk. GWG was assessed using the GA-specific INTERGROWTH-21st and BMI-specific Institute of Medicine (IOM) guidelines. Adverse outcomes included gestational diabetes mellitus (GDM), pregnancy-induced hypertension (PIH), emergency cesarean delivery, low birthweight (LBW), preterm birth, small or large for gestational age (SGA), macrosomia, and birth length or head circumference (HC) <10th or >90th centile.

**Results:**

Inadequate GWG was prevalent, with 53% (*n* = 1767) below the 25th centile of INTERGROWTH-21st standards and 62% (*n* = 2079) below IOM guidelines. Compared with GWG between 25th and 75th centile (*n* = 370), females with GWG <25th centile (*n* = 1767) had a higher odds of SGA [odds ratio (OR) = 2.7, 95% confidence interval (CI): 2.2, 3.4], birth HC < 10th centile (OR: 2.4, 95% CI: 1.8, 3.2), GDM (OR: 1.9, 95% CI: 1.3, 2.7), LBW (OR: 1.9, 95% CI: 1.5, 2.4), and birth length <10th centile (OR: 1.7, 95% CI: 1.4, 2.1). Similarly, females with GWG >75th centile (*n* = 458) had higher odds for emergency cesarean section (OR: 1.7, 95% CI: 1.1, 2.7) and PIH (OR: 1.5, 95% CI: 1.1, 1.9).

**Conclusions:**

Appropriate-for-age-specific GWG between the 25th and 75th centiles standards is associated with reduced adverse outcomes, highlighting the importance of tailored guidelines for optimal maternal and neonatal health.

## Introduction

Measuring maternal weight gain in pregnancy is a long-established clinical practice with major health implications at individual and population levels [1]. However, practice recommendations have varied greatly in the last century. In the past, females were advised to limit gestational weight gain (GWG) to 4.5–6.3 kg to avoid developing pre-eclampsia; in the 1960s, they were advised to gain 9–11 kg to improve birthweight [2]. In 1990, the United States National Academy of Sciences Institute of Medicine (IOM) established GWG guidelines, which were updated in 2009 based on prepregnancy BMI (kg/m^2^) to restrict GWG in females with overweight/obesity [2]. Optimal GWG was defined as 12.5–18.0 kg for females with underweight (<18.5 kg/m^2^), 11.5–16.0 kg for females with normal weight (18.5–24.9 kg/m^2^), 7.0–11.5 kg for females with overweight (25.0–29.9 kg/m^2^), and 5.0–9.0 kg for females with obesity (≥30 kg/m^2^) [3]. Despite being the most followed guidelines, they are not universally accepted [4,5], in part because they were derived from exclusively United States resident data and may, therefore, not be universally applicable, especially in low to middle-income countries [[6], [7], [8], [9]].

Beyond the broad categories of inadequate and excessive GWG based on IOM guidelines, there is a growing recognition that GWG patterns during pregnancy may play a pivotal role in health outcomes [10,11]. The traditional focus on total weight gain throughout pregnancy may overlook the dynamic changes in weight occurring across gestational age periods. Therefore, understanding the specific patterns of GWG at different stages of pregnancy is essential for the management of GWG during pregnancy in a timely and more personalized approach.

The article by Ismail et al. [12] conducted a comprehensive analysis of gestational age-specific GWG patterns in females with normal weight from the Fetal Growth Longitudinal Study within the INTERGROWTH-21st (IG) Project. The strength of their analysis was that it was based on GWG patterns of 8 geographically diverse populations of females who were deemed to be healthy, were followed up during pregnancy with a confirmed healthy newborn at birth, and at 2 y [12].

A remaining gap was to show evidence of the association of these GWG with neonatal and maternal outcomes that can be used for monitoring during pregnancy. To achieve this, we evaluated the association between GWG and maternal and neonatal health outcomes in the INTERBIO-21st study, using GWG between the 25th and 75th centiles from IG as the counterfactual. In the INTERBIO-21st Fetal Study, the primary outcomes were fetal growth patterns and the identification of intrauterine growth restriction and preterm birth, whereas secondary outcomes included maternal risk factors, pregnancy complications, and neonatal anthropometry at birth [13]. On the basis of the available perinatal health outcomes, we have also conducted a comparative analysis of GWG standards set by IG and the recommendations by IOM, particularly focusing on their association with adverse health outcomes in mothers and newborns. We believe the results from our analysis will contribute to discussions regarding what the appropriate GWG thresholds should be.

## Methods

### Study design and participants

Detailed information about each study site, the cohort studied, data management system, and study protocol has previously been published [13] and is available at www.interbio21.org.uk. In brief, the INTERBIO-21st Fetal Study was conducted between 8 February, 2012 and 30 November, 2019, at study sites in Pelotas (Brazil), Nairobi (Kenya), Karachi (Pakistan), Soweto (South Africa), Mae Sot (Thailand), and Oxford (United Kingdom). The aim of this study was to improve the phenotypic characterization of fetal growth restriction and preterm birth syndromes by integrating clinical, biochemical, and molecular data from diverse populations, with a focus on the effects of nutrition and early-life exposures during pregnancy [14].

**FIGURE 1:**
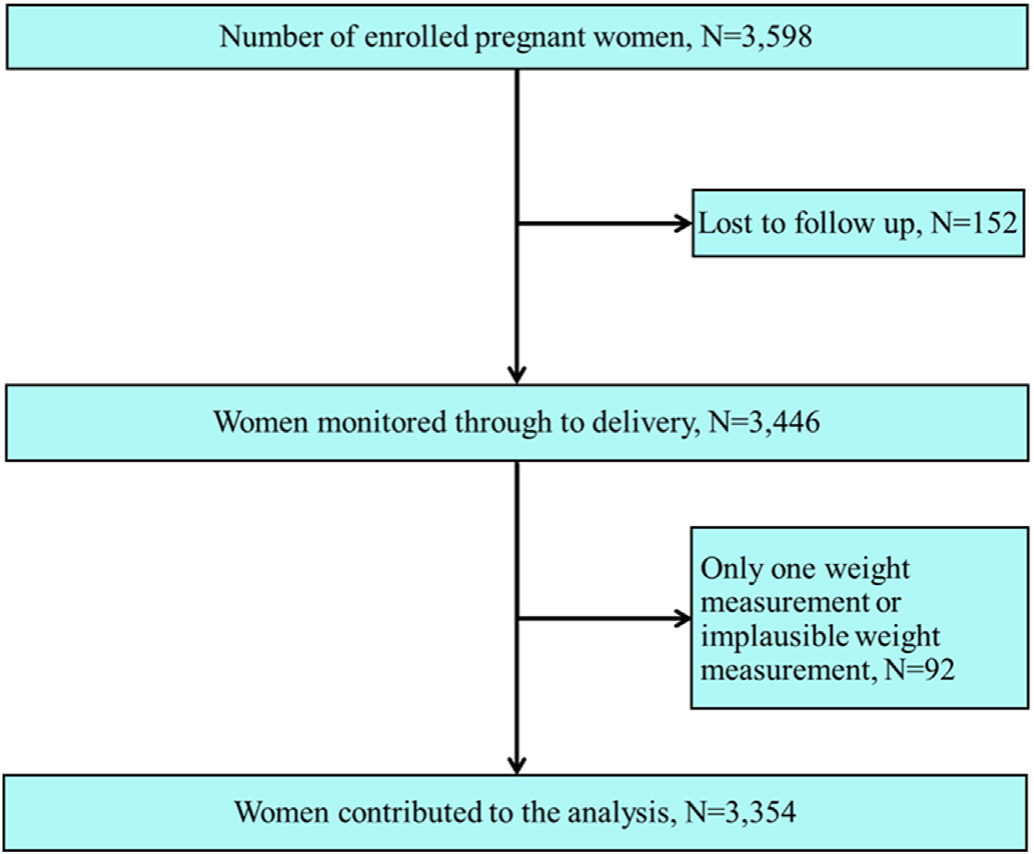
Participant flow chart (*N* = 3354)

We enrolled 3598 females who initiated antenatal care before 14 wk of gestation, as determined by ultrasound dating, regardless of their risk profile for adverse outcomes. After the dating ultrasound scan, the females were followed up every 5 ± 1 wk until delivery. Inclusion criteria included maternal age ≥18 y, BMI <35 kg/m^2^ (for ease of fetal ultrasound scanning), natural conception, and singleton pregnancy. Of the 3598 females, 3446 (96%) were monitored through to delivery, with their children’s health monitored up to age 2. After excluding 92 females with only 1 weight measurement and 50 measures due to implausible GWG (gaining or losing >5 kg/wk) or missing gestational age data, a total of 15,760 measurements for 3354 females (median visits = 5, IQR: 4–5) were analyzed (Figure 1).

### GWG definition

GWG was calculated as the measured weight at each antenatal visit minus the measured weight at the first visit. We generated GWG centiles according to the IG standards using the equation provided by Ismail et al. [12]. We applied the IG GWG centile equation to a new population of 3354 females (15,760 measurements) who participated in the INTERBIO-21st Fetal study from 2012 to 2014. GWG consistently between the 25th and 75th centiles of the IG standard was used as a reference, aligning with the IOM recommendations for females with normal weight [12]. GWG was categorized into: *1*) GWG consistently <25th centile throughout the pregnancy, *2*) GWG <25th centile at any point of the pregnancy, *3*) GWG consistently >75th centile across pregnancy, and *4*) GWG >75th centile at any point of the pregnancy. Total GWG was also calculated as the difference between the last available weight and baseline weight in the first trimester. Using BMI-specific IOM guidelines, we classified GWG into: *1*) less than recommended, *2*) greater than recommended, and *3*) within the recommended range (reference group).

GWG was also categorized based on the IOM guideline. Inadequate GWG was defined as follows: females with underweight (BMI < 18.5 kg/m^2^) gaining <12.5 kg, females with normal weight (BMI 18.5–24.9 kg/m^2^) gaining <11.5 kg, females with overweight (BMI 25–29.9 kg/m^2^) gaining <7 kg, and females with obesity (BMI ≥ 30 kg/m^2^) gaining <5 kg during pregnancy. Conversely, excessive GWG was defined as: females with underweight gaining >18 kg, females with normal weight gaining >16 kg, females with overweight gaining >11.5 kg, and females with obesity gaining >9 kg.

### Maternal/neonatal outcomes

Our analysis is a secondary analysis of data from INTERBIO-21st Fetal Study which mainly focused on understanding how early-life factors—particularly nutrition and adverse intrauterine conditions—affect fetal growth and development. The main outcomes for our analysis were considered any reported maternal or newborn-related adverse outcomes at birth in the INTERBIO-21st Fetal Study. The maternal outcomes for these analyses were pregnancy-induced hypertension (PIH, defined as blood pressure >140/90 without proteinuria); pre-eclampsia (defined as blood pressure >140/90 with proteinuria); gestational diabetes mellitus (GDM, defined as any degree of glucose intolerance with onset or first recognition during pregnancy); preterm birth (PTB, both spontaneous and indicated birth <37 wk of gestation), and mode of delivery (cesarean section due to maternal and neonatal health complexities). The neonatal outcomes were birthweight (kg, within 12 h of birth); gestational age at birth (weeks); low birthweight (LBW, birthweight <2500 g), macrosomia (birthweight > 4000 g); small for gestational age (SGA) or large for gestational age (LGA), birth length <10th or >90th centile, and birth head circumference (HC) <10th or >90th centile (defined, respectively, as <10th and >90th centiles of the IG Newborn Size Standard and Very Preterm Size at Birth Reference) [15,16].

### Covariates

Covariates included study sites (Brazil, Kenya, Pakistan, South Africa, Thailand, and United Kingdom), maternal age (years), maternal education (high school graduate or less compared with college or more), smoking (yes compared with no), alcohol consumption (yes compared with no), nulliparity (yes compared with no), history of hypertension (yes compared with no), and diabetes (yes compared with no) (Supplemental Table 1).

### Ethical approval

The study was approved by the Oxfordshire Research Ethics Committee “C” (reference: 08/H0606/139), the research ethics committees of the participating institutions, and their regional health authorities. All females provided written informed consent.

### Data collection procedures

A comprehensive set of variables was prospectively collected using data collection forms and an electronic data entry system specifically developed for the study. Baseline information, including demographic and nutritional characteristics, medical, maternal morbidity, gynecological, and obstetric history, as well as current pregnancy-related conditions, was included.

Moreover, at each visit, maternal height and weight were measured in duplicate, using a Seca 264 stadiometer and Seca 877 scale (Seca, Germany), respectively. A first-trimester BMI was calculated at the dating scan (that is, upon study entry between 9^+0^ and 13^+6^ wk of gestation). The same standardized methodology was then used to measure maternal weight at every subsequent visit. Thus, the possible ranges after enrollment when weight was measured were 14–18, 19–23, 24–28, 29–33, 34–38, and 39–42 wk of gestation.

### Statistical analyses

Early-pregnancy weight, measured between 9 and <14 wk of gestational age, was used to generate maternal BMI categories in this analysis. Continuous variables were summarized using the mean ± SD or median and IQR, as appropriate, whereas categorical variables were presented as frequencies and percentages.

We conducted multivariable logistic regression to quantify the association between adverse maternal and neonatal outcomes for GWG consistently <25th centile throughout pregnancy and GWG <25th or >75th centile at any point during pregnancy, compared with the reference category of GWG consistently between the 25th and 75th centiles of the IG standard. We could not show the association between perinatal outcomes and GWG consistently >75th centile across pregnancy because only a few females (*n* = 23) gained above the range (Supplemental Table 2). Subsequently, we evaluated the effect of IOM-recommended GWG on adverse maternal and newborn health outcomes.

To ensure robust results, we conducted multiple sensitivity analyses: *1*) comparing GWG IOM recommendations with IG standards; *2*) applying IG standards across BMI categories; *3*) separate analyses for high- and low-income country models to assess their impact; *4*) excluding each study site one at a time to identify any site-specific effects; *5*) validating the IG GWG standard in countries not included in the original IG charts; and *6*) performing trimester-specific analyses. All statistical analyses were performed using the R statistical software (version 4.3.0) and no specific packages were used other than those already available as part of base R.

## Results

### Study population

In this analysis, 3354 females contributed a total of 15,760 weight measurements during pregnancy [median number of visits was 5 (IQR: 4–5)] and 12,405 cumulative gestational age-specific weight gain. The baseline sociodemographic characteristics of these females are summarized in Table 1, with details by study site in Supplemental Table 3. The median age was 30 y (IQR: 26–34); most females were married (85%) and 71% had at least a secondary education. The contribution from the 6 study sites to the total population varied between 12% (Pelotas, *n* = 400) and 20% (Oxford, *n* = 660).

**TABLE 1:** Baseline sociodemographic characteristics of 3354 females who participated in the INTERBIO-21st Fetal Study.

Characteristic	Overall (*N* = 3354)
Study sites, *n* (%)	
Brazil	400 (11.9%)
Kenya	586 (17.5%)
Pakistan	535 (16.0%)
South Africa	607 (18.1%)
Thailand	566 (16.8%)
United Kingdom	660 (19.7%)
Mother’s age (y), median (IQR)	30 (26–34)
Father’s age (y), median (IQR)	32 (28–37)
Marital status, *n* (%)	
Married/cohabiting	2857 (85.2%)
Single	488 (14.5%)
Separated/divorced	6 (0.2%)
Widowed	3 (0.1%)
Mother’s education level, *n* (%)	
No school attended	153 (4.6%)
Primary	337 (10.0%)
Professional/technical training	463 (13.8%)
Secondary	1114 (33.2%)
University	1287 (38.4%)
Mother’s occupation, *n* (%)	
Clerical support, service, or sales	472 (14.1%)
Housework	997 (29.7%)
Managerial/professional/technical	1012 (30.2%)
Skilled manual work	168 (5.0%)
Unskilled manual work	227 (6.8%)
Student	98 (2.9%)
Other	379 (11.3%)
Mother’s height (cm), median (IQR)	160 (155–165)
Mother’s weight (kg), median (IQR)	63 (54–72)
Mother’s BMI (kg/m^2^), median (IQR)	24 (21–28)
Mother’s BMI status, *n* (%)	
Underweight (BMI < 18.5 kg/m^2^)	170 (5.1%)
Normal weight (BMI 18.5–24.9 kg/m^2^)	1686 (50.3%)
Overweight (BMI 25–29.9 kg/m^2^)	1026 (30.6%)
Obese (BMI ≥ 30 kg/m^2^)	471 (14.0%)
Nulliparity, *n* (%)	992 (29.6)
Smoking, *n* (%)	169 (5.1)
Alcohol consumption, *n* (%)	80 (2.4)

**FIGURE 2:**
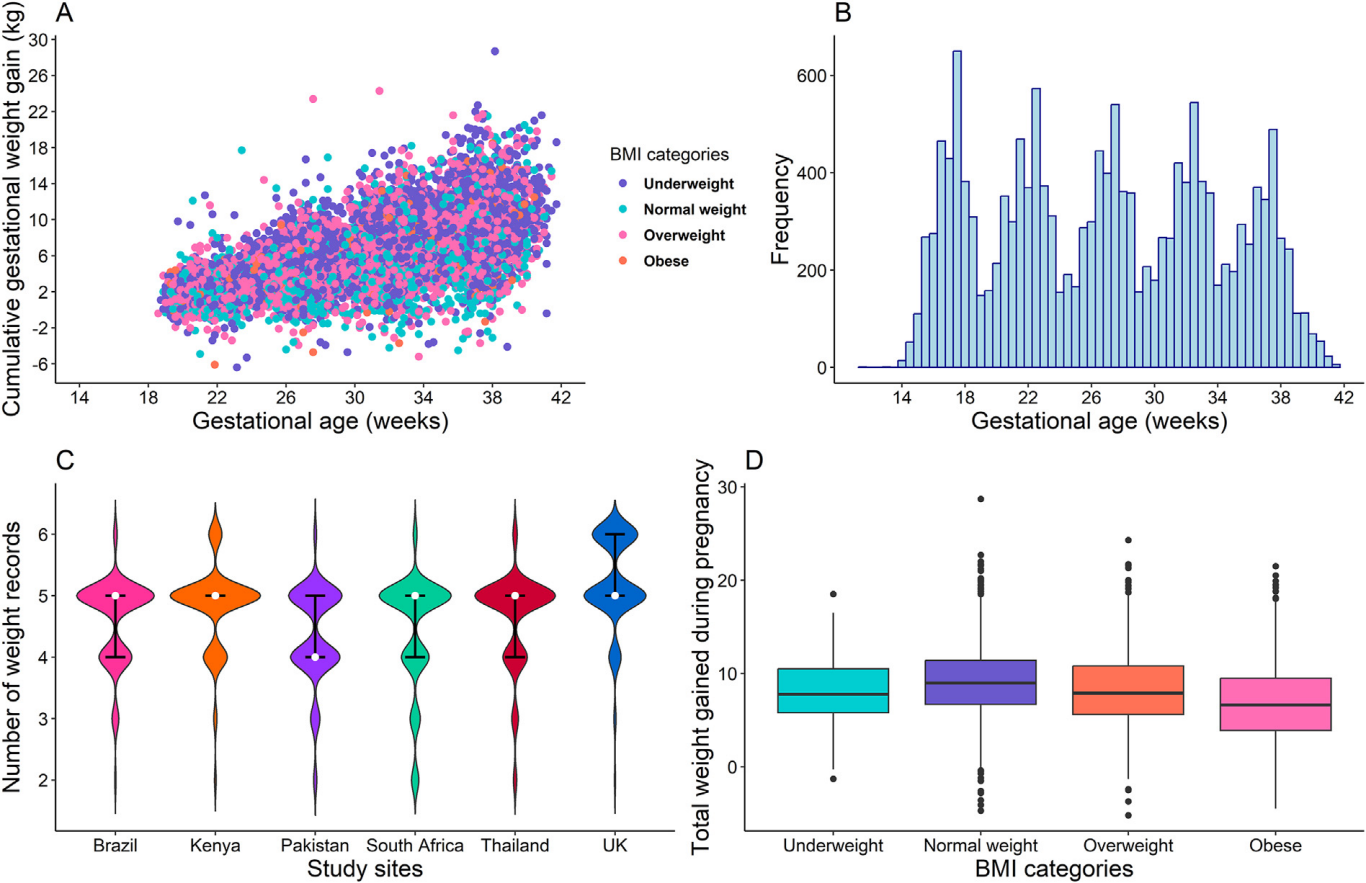
(A) A scatter plot of the distribution of maternal cumulative gestational weight gain by BMI categories (*n* = 3354), (B) a distribution plot of gestational age at which weight was measured for the INTERBIO-21st pregnant females, (C) the distribution of weight records by study sites, and (D) distribution of total weight gain during pregnancy by BMI status.

Half the females had normal weight (BMI 18.5–24.9 kg/m^2^); 31% were overweight (BMI 25–29.9 kg/m^2^), 14% obese (BMI ≥ 30 kg/m^2^), and 5% underweight (BMI < 18.5 kg/m^2^) (Table 1). Overall, the median total weight gain during pregnancy was 8 kg (IQR: 6–11) and was highest (8.5 kg, IQR: 6.2–11.1) among females with normal weight and lowest (6.4 kg, IQR: 3.5–9.1) among females with obesity (Table 2 and Figure 2) [17,18].

Maternal complications included PIH (4.4%, *n* = 144), pre-eclampsia (3.5%, *n* = 59), and GDM (6.2%, *n* = 204) (Table 2). Prevalence of PIH and GDM was ≥11% among obese and was around 4% among females with normal weight (Table 2). Around 21% (*n* = 714) of females had an emergency cesarean section due to maternal or fetal health issues. The mean gestational age at birth was 39 wk (IQR: 38–40) and 12% (*n* = 395) of pregnancies resulted in PTB (gestational age at birth <37 gestational weeks). The median birthweight was 3100 g (IQR: 2772–3434); 12% LBW (*n* = 389), 14% (*n* = 466) were SGA, and 6.8% (*n* = 224) LGA (Table 2). Site-specific baseline sociodemographic characteristics and perinatal events are presented in Supplemental Tables 3 and 4.

**TABLE 2:** Maternal and neonatal health outcomes by early-pregnancy BMI categories in the INTERBIO-21st Fetal Study.

Characteristic	Overall (*N* = 3354)	Underweight (*N* = 170)	Normal weight (*N* = 1686)	Overweight (*N* = 1026)	Obese (*N* = 471)
Total gestational weight gain (kg), median (IQR)	7.9 (5.5–10.7)	7.6 (5.4–10.0)	8.5 (6.2–11.1)	7.7 (5.2–10.6)	6.4 (3.5–9.1)
GWG <25th centile throughout pregnancy	1767 (52.7%)	94 (55.3%)	802 (47.6%)	558 (54.4%)	312 (66.2%)
GWG >75th centile throughout pregnancy	23 (0.7%)	1 (0.6%)	6 (0.4%)	10 (1.0%)	6 (1.3%)
GWG less than IOM lower limit	2079 (62.0%)	155 (91.2%)	1309 (77.6%)	441 (43.0%)	174 (36.9%)
GWG above IOM upper limit	368 (11.0%)	1 (0.6%)	55 (3.3%)	193 (18.8%)	119 (25.3%)
Gestational age at delivery (wk), median (IQR)	39.3 (38.1–40.3)	39.1 (37.9–40.1)	39.4 (38.3–40.3)	39.1 (37.9–40.3)	38.7 (37.6–40.1)
Pre-eclampsia, n (%)	59 (1.8%)	2 (1.2%)	20 (1.2%)	22 (2.1%)	15 (3.2%)
Pregnancy-induced hypertension, *n* (%)	144 (4.3%)	6 (3.5%)	57 (3.4%)	47 (4.6%)	34 (7.2%)
Gestational diabetes, *n* (%)	204 (6.1%)	0 (0%)	59 (3.5%)	89 (8.7%)	56 (11.9%)
Mode of delivery,^1^*n* (%)					
Assisted breech	4 (0.1%)	0 (0.0%)	4 (0.2%)	0 (0.0%)	0 (0.0%)
Cesarean section	1306 (38.9%)	24 (14.1%)	509 (30.2%)	502 (48.9%)	271 (57.5%)
Vaginal assisted	154 (4.6%)	7 (4.1%)	97 (5.8%)	43 (4.2%)	7 (1.5%)
Vaginal spontaneous	1819 (54.3%)	132 (77.6%)	1034 (61.3%)	464 (45.2%)	189 (40.1%)
Emergency cesarean delivery, *n* (%)	714 (21.3%)	16 (9.4%)	310 (18.4%)	264 (25.7%)	124 (26.3%)
Preterm birth, *n* (%)	395 (11.8%)	24 (14.1%)	163 (9.7%)	133 (13.0%)	75 (15.9%)
Newborn sex: male, *n* (%)	1698 (50.6%)	76 (44.7%)	864 (51.2%)	527 (51.4%)	230 (48.8%)
Birthweight (grams)^1^, median (IQR)	3100 (2773–3434)	2858.8 (2524–3101)	3076.3 (2778–3375)	3180.0 (2820–3506)	3125.0 (2781–3484)
Low birth weight, *n* (%)	389 (11.6%)	39 (22.9%)	185 (11.0%)	115 (11.2%)	50 (10.6%)
Macrosomia, *n* (%)	118 (3.5%)	1 (0.6%)	54 (3.2%)	45 (4.4%)	18 (3.8%)
Small for gestational age (SGA),^2^*n* (%)	466 (13.9%)	43 (25.3%)	255 (15.1%)	117 (11.4%)	51 (10.8%)
Large for gestational age (LGA),^2^*n* (%)	224 (6.7%)	3 (1.8%)	84 (5.0%)	90 (8.8%)	47 (10.0%)
Birth length (cm),^1^ median (IQR)	48.8 (47.3–50.0)	48.0 (46.6–49.5)	48.7 (47.3–50.0)	49.0 (47.5–50.2)	49.0 (47.3–50.3)
Birth length <10th centile,^2^*n* (%)	432 (12.9%)	36 (21.2%)	232 (13.8%)	120 (11.7%)	44 (9.3%)
Birth length >90th centile,^2^*n* (%)	296 (8.8%)	6 (3.5%)	123 (7.3%)	114 (11.1%)	53 (11.3%)
Birth head circumference (cm),^1^ median (IQR)	33.9 (32.8–34.8)	32.8 (31.9–33.7)	33.8 (32.7–34.7)	34.1 (33.2–35.0)	34.0 (33.1–35.0)
Birth head circumference <10th centile,^2^*n* (%)	300 (8.9%)	44 (25.9%)	185 (11.0%)	48 (4.7%)	23 (4.9%)
Birth head circumference >90th centile,^2^*n* (%)	410 (12.2%)	4 (2.4%)	164 (9.7%)	154 (15.0%)	88 18.7%)

Abbreviations: GWG, gestational weight gain; IOM, Institute of Medicine.

1 Birthweight was missing for 112 (3.3%), birth length was missing for 168 (5.0%) and birth head circumference was missing for 155 (4.6%), mode of delivery information was missing for 42 females.

2 SGA, LGA, length<10th centile, length>90th centile, head circumference<10th centile, head circumference>90th centile were defined, respectively, as <10th and >90th centiles of the INTERGROWTH-21st Newborn Size Standard and Very Preterm Size at Birth Reference [17,18].

### GWG trajectories

Throughout pregnancy, 1767 females (53%) gained consistently below the 25th centile of the IG standards. A total of 2801 females (8226 observations) had ≥1 GWG below 25th centile IG standard at some point of the pregnancy (Supplemental Table 2). Around 11% (*n* = 367) of females gained continuously below the third centile IG standard throughout the pregnancy. Of all GWG records, 243 (7.2%) observations were above the 75th centile at some point during the pregnancy. Around 11% of females (*n* = 370) gained consistently between the 25th and 75th centile of IG standard throughout pregnancy. Further analyses on GWG patterns by early-pregnancy BMI categories demonstrated that around two-thirds of females with obesity (*n* = 312) gained consistently below 25th centile GWG IG standard and was 55%, 48%, and 54% for females with underweight, normal weight, and overweight, respectively (Supplemental Table 2).

On the basis of BMI-specific IOM guidelines for GWG, ∼62% (*n* = 2079) of females gained less weight than recommended. Overall, 11% (*n* = 368) of females exceeded the upper GWG limits, whereas 27% (*n* = 906) gained weight within the recommended range (Supplemental Table 2).

Across the 6 study sites, ≥35% of females consistently gained below the 25th centile throughout pregnancy, with the highest prevalence in Pakistan (66%) and the lowest in the United Kingdom (35%). Additionally, ≥46% of females in all sites gained less than the IOM standard (Supplemental Table 4).

### Associations between gestational age-specific weight gain (WG) centiles and maternal outcomes

**FIGURE 3:**
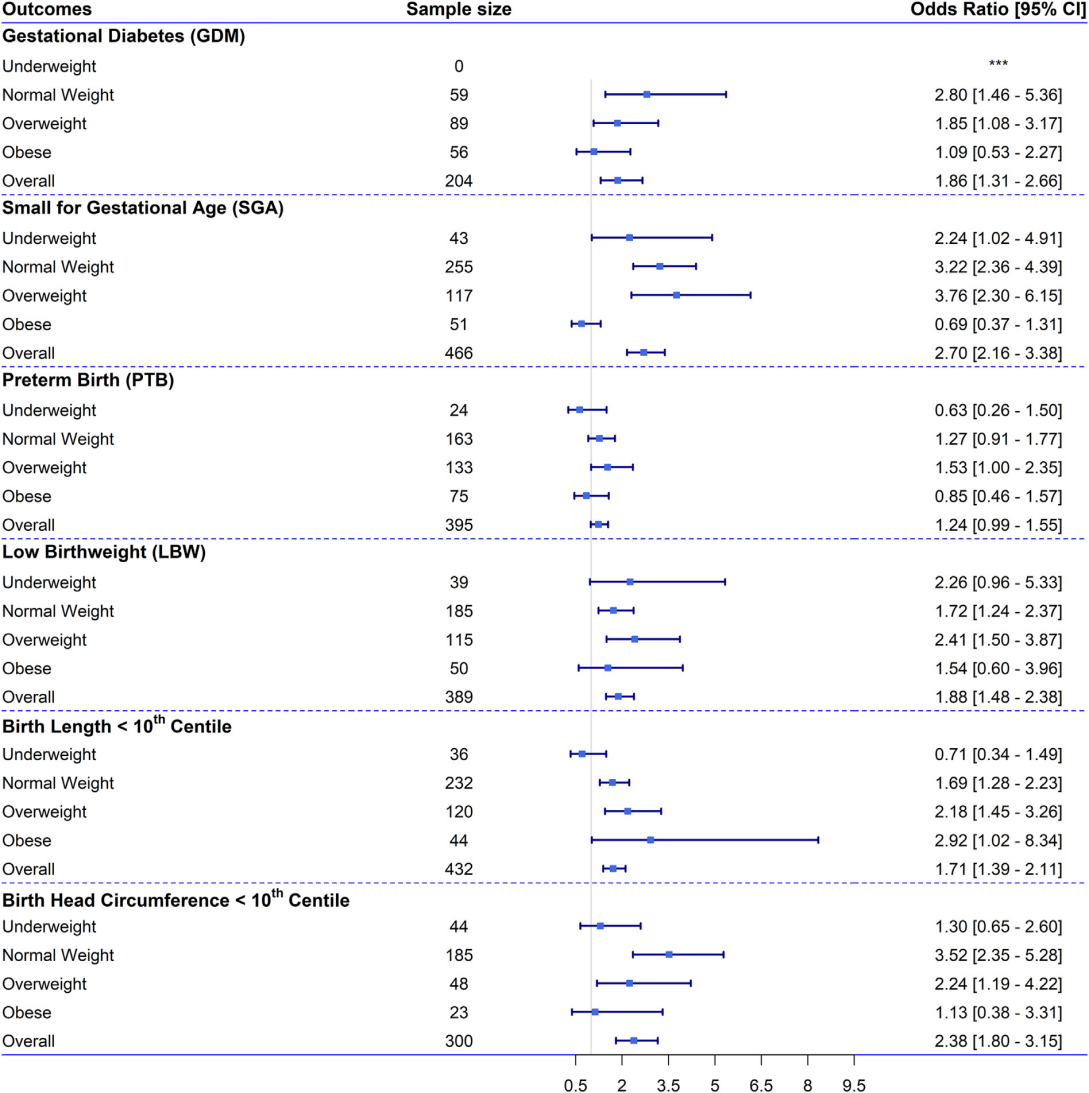
Odds ratios (ORs) with 95% confidence intervals (CIs) for the association between perinatal health outcomes and gestational age-specific weight gain consistently below the 25th centile in reference to gaining weight consistently between 25th and 75th centile throughout pregnancy of the INTERGROWTH-21st (IG) standard, in the overall sample and across subgroups of females with different levels of BMI. Note: ORs with 95% CIs for the association between perinatal health outcomes [gestational diabetes mellitus (GDM), small for gestational age (SGA), preterm birth (PTB), low birthweight (LBW), birth length <10th centile, and birth head circumference <10th centile] and gestational weight gain (GWG) consistently below the 25th centile throughout pregnancy of the IG standard, in the overall sample and across maternal BMI categories. The ORs and 95% CIs are shown for the association between GWG consistently below the 25th centile of the gestational age-specific IG standard throughout pregnancy (*n* = 1766) and adverse perinatal health outcomes. These associations were analyzed using multivariable regression models adjusted for possible covariates. The associations are shown across early-pregnancy BMI categories: underweight (*n* = 170), normal weight (*n* = 1686), overweight (*n* = 1026), and obese (*n* = 471). The frequency of females gaining GWG < 25th centile IG standard was 802 for normal weight, 558 for overweight, and 312 for females with obesity. The reference group includes females gaining gestational weight consistently between the 25th and 75th centiles of the IG standard during pregnancy (overall, *n* = 370; underweight, *n* = 20; normal weight, *n* = 216; overweight, *n* = 102; obese, *n* = 32). For each outcome, the sample size represents the total number of females who experienced the outcome in each BMI category and in the overall sample.

**FIGURE 4:**
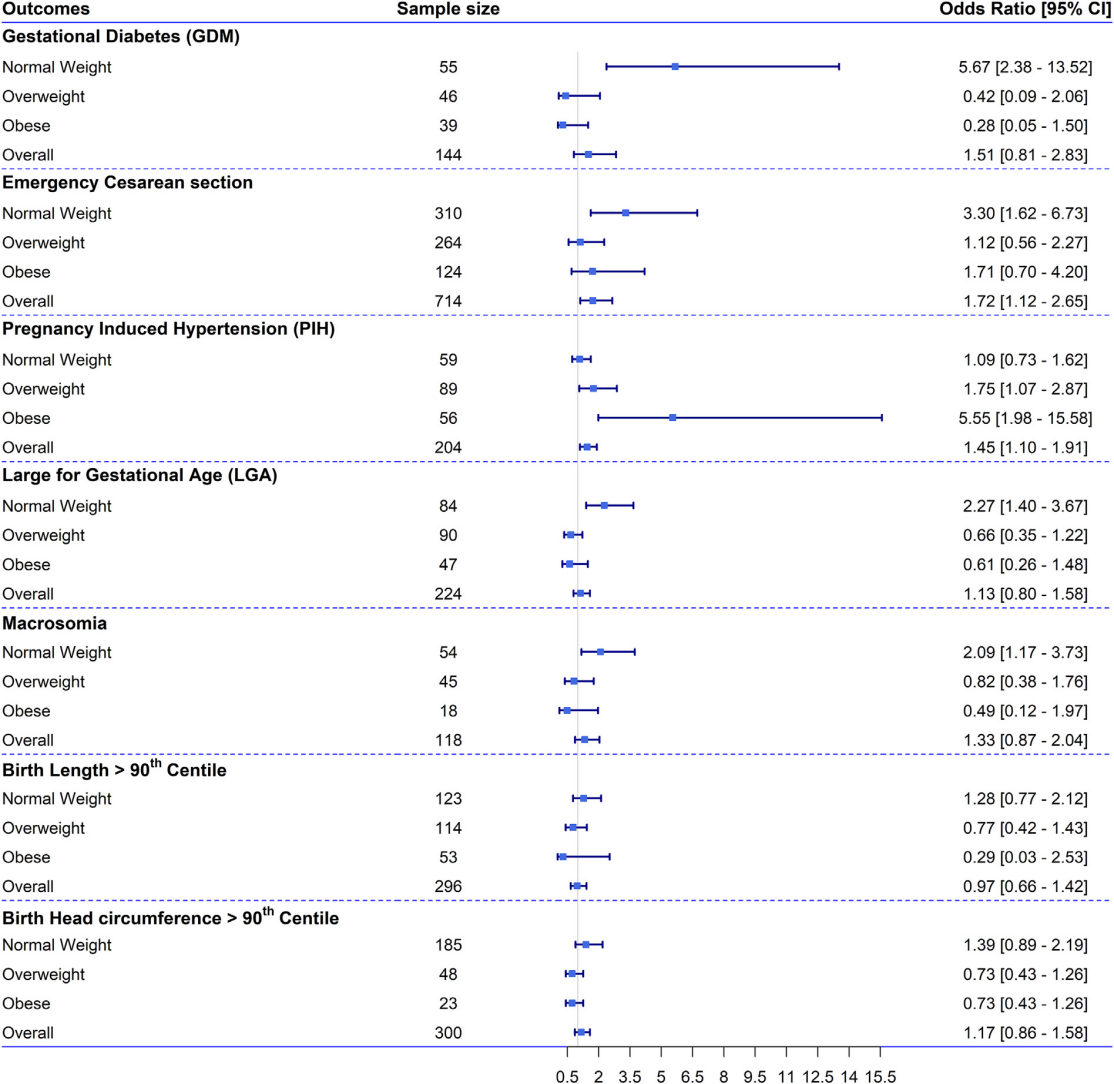
Odds ratios (ORs) with 95% confidence intervals (CIs) for the association between perinatal health outcomes and gestational age-specific weight gain above the 75th centile at any point of pregnancy in reference to gaining weight consistently between 25th and 75th centile throughout pregnancy of the INTERGROWTH-21st (IG) standard, in the overall sample and across subgroups of females with different levels of across maternal BMI. Note: ORs with 95% CIs for the association between perinatal health outcomes [gestational diabetes mellitus (GDM), small for gestational age (SGA), preterm birth (PTB), low birthweight (LBW), birth length <10th centile, and birth head circumference <10th centile] and gestational weight gain (GWG) above the 75th centile at any point of pregnancy of the IG standard, in the overall sample and across maternal BMI categories. The ORs and 95% CIs are shown for the association between GWG above the 75th centile of the gestational age-specific IG standard at any point of pregnancy (*n* = 243) and adverse perinatal health outcomes. These associations were analyzed using multivariable regression models adjusted for possible covariates. The associations are shown across early-pregnancy BMI categories: normal weight (*n* = 1686), overweight (*n* = 1026), and obese (*n* = 471). The association could not be shown for females with underweight due to the small sample size. The frequency of females gaining GWG >75th centile IG standard at any point of pregnancy was 5 for underweight, 129 for normal weight, 79 for overweight, and 30 for females with obesity. The reference group includes females gaining gestational weight between the 25th and 75th centiles of the IG standard during pregnancy (overall, *n* = 370; underweight, *n* = 20; normal weight, *n* = 216; overweight, *n* = 102; obese, *n* = 32). For each outcome, the sample size represents the total number of females who experienced the outcome in each BMI category and in the overall sample.

On the basis of our multivariable regression analysis for the overall sample and across the 4 BMI groups (FIGURE 3, FIGURE 4, Supplemental Figure 1, 2, and 3), we observed a significant association between GWG centiles and maternal outcomes. Compared with the reference group with GWG consistently between the 25th and 75th centiles, the odds of developing GDM almost doubled [odds ratio (OR) = 1.9; 95% CI: 1.3, 2.7] for females consistently gaining <25th centile of the IG standard. The odds of GDM were around 3-fold higher (OR: 2.8; 95% CI: 1.5, 5.4) for females with normal weight compared with other females with other BMI status (Figure 3). A similar association was observed for females gaining <25th centile at any point of pregnancy (Supplemental Figure 1).

The odds of PIH were 1.5 times higher (OR: 1.45; 95% CI: 1.10, 2.91) among females with GWG >75th centile at any point during pregnancy, with an even greater odds among females with obesity (OR: 5.6; 95% CI: 2.0, 15.6) (Figure 4). Similarly, the overall odds of an emergency cesarean delivery were 1.7 times higher (OR: 1.72; 95% CI: 1.1, 2.7), and the odds tripled for females with normal weight (OR: 3.30; 95% CI: 1.7, 6.7) when GWG exceeded the 75th centile. No significant association was found between GWG and cesarean delivery across other BMI categories.

### Associations between gestational age-specific WG centiles and neonatal outcomes

We observed a significant association between gestational age-specific IG GWG centiles and neonatal outcomes (FIGURE 3, FIGURE 4, Supplemental Figures 1, 2, 4, 5, 6, 7, and 8, Table 3). Overall, the odds was around 2 times higher for: SGA (OR: 2.70; 95% CI: 2.16, 3.38), birth length <10th centile (OR: 2.38; 95% CI: 1.80, 3.15), LBW (OR: 2.03; 95% CI: 1.67, 2.48), and birth HC <10th centile (OR: 1.96; 95% CI: 1.59, 2.44) when GWG was consistently <25th centile throughout pregnancy compared with the reference group (Figure 3 and Table 3).

**TABLE 3:** Adjusted odds ratio with 95% confidence interval (CI) of maternal and neonatal health outcomes for gestational weight gain (GWG) INTERGROWTH-21st centiles and IOM recommendation for 3354 females.

Predictors	GWG < 25th centile throughout pregnancy (*N* = 1767)			GWG < 25th centile at any point of pregnancy (*N* = 2801)			GW < BMI-specific IOM standard (*n* = 2079)		
	Odds ratios	95% CI	*P* value	Odds ratios	95% CI	*P* value	Odds ratios	95% CI	
Gestational diabetes, *n* = 204	1.86	1.31, 2.66	0.001	1.78	1.26, 2.53	0.001	2.19	1.47, 3.25	<0.001
Preterm birth, *n* = 395	1.24	0.99, 1.55	0.064	1.13	0.91, 1.41	0.257	2.73	2.02, 3.68	<0.001
Low birthweight, *n* = 389	1.88	1.48, 2.38	<0.001	1.63	1.29, 2.06	<0.001	2.68	1.95, 3.68	<0.001
Small for gestational age, *n* = 466	2.70	2.16, 3.38	<0.001	2.31	1.85, 2.88	<0.001	1.84	1.39, 2.43	<0.001
Birth length< 10th centile, *n* = 432	1.71	1.39, 2.11	<0.001	1.53	1.25, 1.88	<0.001	1.57	1.19, 2.08	0.002
Birth head circumference< 10th centile, *n* = 300	2.38	1.80, 3.15	<0.001	2.09	1.59, 2.75	<0.001	2.20	1.49, 3.25	<0.001

Predictors	GWG >75th centile throughout pregnancy (*N* = 23)			GWG >75th centile at any point of pregnancy (*N* = 243)			GW > IOM standard (*n* = 368)		
	Odds ratios	95% CI	*P* value	Odds ratios	95% CI	*P* value	Odds ratios	95% CI	*P* value
Pregnancy-induced hypertension, *n* = 144	∗∗∗			1.72	1.12, 2.65	0.013	1.61	0.95, 2.74	0.076
Gestational diabetes, *n* = 204	∗∗∗			1.51	0.81, 2.83	0.193	1.08	0.56, 2.09	0.822
Emergency C-section, *n* = 714	∗∗∗			1.45	1.10, 1.91	0.009	1.26	0.92, 1.73	0.145
Large for gestational age, *n* = 224	∗∗∗			1.13	0.80, 1.58	0.490	2.07	1.40, 3.06	<0.001
Macrosomia, *n* = 118	∗∗∗			1.33	0.87, 2.04	0.195	1.72	1.05, 2.83	0.032
Birth length > 90th centile, *n* = 296	∗∗∗			0.97	0.66, 1.42	0.863	1.42	0.95, 2.13	0.089
Birth head circumference> 90th centile, *n* = 410	∗∗∗			1.17	0.86, 1.58	0.323	1.05	0.75, 1.48	0.760

The odds ratios (ORs) and 95% confidence intervals (CIs) are shown for the association between gestational weight gain (GWG) below and above INTERGROWTH-21st (IG) standard during pregnancy (*n* = 1767) and adverse perinatal outcomes. These associations were analyzed using multivariable regression models adjusted for possible covariates. IG standards are gestational age specific. IOM recommended range: underweight (12.5–18 kg), normal weight (11.5–16 kg), overweight (7–11.5 kg), and obese (5–9 kg) during pregnancy. According to the IOM guidelines, GWG below the IOM standard is defined as follows: females with underweight (<18.5 kg/m^2^) gaining <12.5 kg, females with normal weight (18.5–24.9 kg/m^2^) gaining <11.5 kg, females with overweight (25–29.9 kg/m^2^) gaining <7 kg, and females with obesity (≥30 kg/m^2^) gaining <5 kg during pregnancy. According to the IOM guidelines, GWG above the IOM standard is defined as follows: females with underweight (<18.5 kg/m^2^) gaining >18 kg, females with normal weight (18.5–24.9 kg/m^2^) gaining >16 kg, females with overweight (25–29.9 kg/m^2^) gaining >11.5 kg, and females with obesity (≥30 kg/m^2^) gaining >9 kg during pregnancy. ∗∗∗We could not explore any association between maternal outcomes and females gaining consistently >75th centile GWG IG standard because of small sample (*n* = 23).

Abbreviation: IOM, Institute of Medicine.

For consistent GWG <25th centile, the odds of SGA doubled among females with underweight (OR: 2.24; 95% CI: 1.02, 4.91), was 3-fold higher (OR: 3.22; 95% CI: 2.36, 4.39) for normal weight, and almost 4 times higher for females with overweight (OR: 3.76; 95% CI: 2.30, 6.15) (Figure 3). Females with overweight had higher odds of LBW (OR: 2.41; 95% CI: 1.50, 3.87), whereas both females with overweight (OR: 2.18; 95% CI: 1.45, 3.26) and obesity (OR: 2.92; 95% CI: 1.02, 8.34) had increased odds of birth length <10th centile (Figure 3). For birth HC <10th centile, the odds were double in the overall sample (OR: 2.38; 95% CI: 1.80, 3.15) and among females with overweight (OR: 2.24; 95% CI: 1.19, 4.22) with GWG consistently <25th centile compared with the reference group. The odds were 3.5 times higher (OR: 3.52; 95% CI: 2.35, 5.28) among normal weight females gaining <25th centile IG standard throughout pregnancy. However, we did not observe any significant association between GWG consistently <25th centile and preterm birth, SGA, LBW, and birth HC<10th centile among females with obesity (Figure 3).

In contrast, females with normal weight gaining >75th centile GWG at any point of pregnancy compared with the reference GWG, the odds doubled for LGA (OR: 2.27; 95% CI: 1.40, 3.67) and macrosomia (OR: 2.09; 95% CI: 1.17, 3.73). However, we did not observe any significant association between GWG and birth length >90th centile and birth HC >90th centile in the overall sample and across BMI categories (Figure 4).

### Comparison between IOM and IG GWG standards

We compared the BMI-specific IOM and gestational age-specific IG GWG standards in the overall sample and across BMI categories (FIGURE 2, FIGURE 3, FIGURE 4, Table 3, Supplemental Figure 2A-2B). The distribution of the adverse health outcomes for females gaining <25th centile of IG standard compared with females gaining below the IOM recommendations was very similar (Supplemental Table 5). In the overall sample, for GDM, LBW, SGA, birth length, and HC <10th centile, the odds were almost double when GWG was consistently below the 25th centile throughout pregnancy or total weight during pregnancy was less than the BMI-specific IOM recommended lower limit. For some outcomes compared with IOM standards, the IG standards predicted higher risks. For instance, odds of SGA (OR: 2.70, 95% CI: 2.16, 3.38), birth length <10th centile (OR: 1.71, 95% CI: 1.39, 2.11) and birth HC <10th centile (OR: 2.38; 95% CI: 1.80, 3.15) for females gaining <25th centile IG standard, whereas the OR was 1.84 (95% CI: 1.39, 2.43), 1.57 (95% CI: 1.19, 2.08) and 2.20 (95% CI: 1.49, 3.25) times, respectively, for females gaining total weight below the IOM standard. However, the IOM standard predicted that odds of GDM, PTB, and LBW were higher compared with the IG standard predictions (Table 3).

Females gaining >75th centile of IG standard at any point of pregnancy compared with the reference had a higher odd of PIH (OR: 1.72; 95% CI: 1.12, 2.65) and emergency cesarean section (OR: 1.45; 95% CI: 1.10, 1.91). However, the odds of LGA increased by 2.07 times (OR: 2.07; 95% CI: 1.40, 3.06) and macrosomia by 1.72 times (95% CI: 1.05, 2.83) for females exceeding the IOM recommended upper limit (Supplemental Figure 2A-2B).

When we implemented IOM and IG GWG standards across early-pregnancy BMI categories, we observed that for females with normal weight the association between IG and IOM standards with maternal and neonatal health outcomes was similar (FIGURE 3, FIGURE 4, Supplemental Figures 2a-2b).

### Sensitivity analysis

The association between GWG IG standards and adverse health outcomes based on high-income and low-income countries is presented in Supplemental Figure 9. Overall, the patterns were similar across income levels, but the odds of LBW were higher in low-income countries for females consistently gaining below the 25th centile. In contrast, the odds of SGA, birth length, and HC<10th centile were higher in high-income countries for females gaining below the 25th centile. Site-specific analysis (Supplemental Figure 10) showed similar trends in perinatal outcomes across countries. Validation of IG standards in noncontributing countries (Supplemental Figure 11) confirmed consistent risk prediction of adverse outcomes across all sites, though the association with GDM was significant only in countries contributing to the IG study.

A trimester-based analysis (Supplemental Table 6) revealed that adverse outcomes were more strongly associated with GWG deviations in the third trimester. Consistently gaining below the 25th centile in the third trimester significantly (*P*<0.05) increased the odds of LBW (OR: 1.96), SGA (OR: 2.54), birth length <10th centile (OR: 1.66), and birth HC <10th centile (OR: 2.40). Gaining above the 75th centile in the third trimester was significantly associated with PIH (OR: 2.52) and emergency C-section (OR: 1.84), but not with other perinatal outcomes.

## Discussion

### Principal findings

We have demonstrated that inadequate GWG (that is, GWG consistently <25th centile of IG standard throughout pregnancy) was associated across pregnancy with almost a doubling of odds for GDM, LBW, SGA, birth length <10th centile, and birth HC <10th centile. GWG >75th centile of IG standard at any point of pregnancy was associated with ≥1.5 times odds for PIH and emergency cesarean delivery. Our findings complement the well-established U-shaped relationship between inadequate or excessive GWG and adverse outcomes [7,9,19].

### Comparison with other studies

In the IOM’s 2009 examination of how their guidelines were being used, GWG was mostly reported as a total, or ratio of the total, weight gain during pregnancy [20]. However, total weight gain is prone to bias as it is a function of gestational age, as indeed are the outcomes PTB, SGA, and LGA [2]. Weekly rate of GWG has been used to quantify GWG trajectories but the assumption of linear weight gain during pregnancy is incorrect [19].

Comparisons with other GWG trajectory approaches are difficult because the studies: *1*) lack clear guidance on GWG thresholds; *2*) vary widely by study population and design, and *3*) use a range of different statistical methods to plot trajectories. In this study, we used the GWG standards from IG to determine the optimal GWG centiles using data from multinational study sites, a method highlighted in recent research as a potential global standard for GWG monitoring [8,9,[21], [22], [23], [24]].

Nevertheless, the weight gain observed between the 25th and 75th centiles at term in the Fetal Growth Longitudinal Study (10.9–17.9 kg) was comparable with the IOM recommendation for females with normal weight (11.5–16.0 kg) and aligned with optimal GWG reported in a meta-analysis of 196,670 participants from 25 cohort studies (1989–2015) [25]. A recent study in the United States found that a GWG associated with no more than a 5% increase in risk ranged from 11.2 to 15.3 kg at 40 wk [26]. Our study's significance lies in defining GWG, capturing its nonlinear trajectory, unlike total weight gain measures.

In this secondary analysis across 6 multinational sites, 53% of females gained below the IG standard, and 62% fell short of the IOM guidelines, reflecting trends seen in other studies [[7], [8], [9],^21^,^22^]. Only 11% exceeded the IOM standards, and just 1% surpassed the IG standards. Excessive weight gain was more prevalent in the United Kingdom (IOM = 20%, IG = 12%) compared with other sites. Similarly, a systematic review of over 1 million females found that those in the United States and Europe had a higher prevalence of excessive weight gain than females in Asia [7].

Consistent with previous research, inadequate GWG (<25th centile IG or <IOM) and excessive GWG (>75th centile IG or >IOM) were associated with adverse outcomes, including LBW, SGA, and smaller birth length/HC [[7], [8], [9],[27], [28], [29]]. Nonetheless, The IG standards showed comparable but less pronounced effects than IOM guidelines [9].

We observed ≥1.9-fold odds of GDM with inadequate GWG. However, a retrospective cohort study in China involving 13,366 pregnant females found an increased risk of GDM for those with GWG above IG standards (OR: 1.27; 95% CI: 1.18, 1.37) and IOM standards (OR: 1.22; 95% CI: 1.13, 1.32) [23]. In our study, the odds of GDM increased by almost 6 times only among females with normal weight for gaining >75th centile at any point of pregnancy. A key methodological difference is that the Chinese study measured GWG before GDM development, whereas our study considered GWG over the entire pregnancy. The higher GDM risk with inadequate GWG in our study might be due to females with GDM experiencing weight loss from following a calorie-restricted diet as part of their treatment.

Our study found that the IOM guidelines were strongly associated with LGA and macrosomia in females with overweight, whereas the IG standards showed no significant association, likely due to the smaller proportion of females exceeding the 75th centile. In contrast, a United States cohort study reported that GWG below the IOM recommendations in females with overweight and obesity did not increase risks for adverse outcomes and was associated with reduced complications such as pre-eclampsia and postpartum weight retention [30]. These findings indicate a need to reconsider the upper weight limits set by the IOM guidelines for these BMI groups. However, the diverse outcomes observed across study sites in our research underscore the importance of further investigating GWG recommendations across BMI categories to ensure they are both population-specific and globally applicable [9,21,30]

### Strengths and limitations of study

Gestational weight gain has predominantly been studied in high-income countries, with limited data from diverse international populations [17]. In contrast, our prospective, longitudinal study took a more global approach, gathering maternal and neonatal data—including an average of 5 weight measurements per woman— across 6 countries on 3 continents using a standardized protocol. We observed greater variation in inadequate and excessive GWG distribution per IOM standards compared with IG standards, suggesting regional differences may influence GWG patterns.

Using data from the INTERBIO-21st study, which included comprehensive maternal demographics, health behaviors, and birth outcomes, we thoroughly assessed GWG's association with maternal/neonatal health outcomes, controlling for confounding factors. It is noteworthy that many previous studies have been constrained by limited data availability, often resulting in either overestimation or underestimation of associations.

In addition, we assessed the potential for GWG trajectories to predict maternal/neonatal outcomes across different early-pregnancy BMI categories, multinational sites, and different phases of pregnancy. The reference GWG was derived from the IG Project [18], conducted in 8 geographically diverse regions worldwide using the same study protocol but with healthy, well-nourished participants who met the WHO prescriptive criteria for generating international growth standards [31].

In fact, the implementation of our findings supports global initiatives, such as the WHO’s efforts [32], to develop comprehensive GWG guidelines across various BMI ranges and contexts. By aligning our gestational age-specific insights with these efforts, we can facilitate universally applicable GWG guidelines, offering healthcare providers a consistent framework for supporting pregnant individuals.

However, the study has several limitations. Despite a large overall sample, the 6 study sites are not representative of the broader population. For instance, the Maternal and Child Health and Nutrition in Acre (MINA-Brazil) population-based birth cohort in Cruzeiro do Sul, Acre State observed around 65% of females with insufficient weight gain based on the IOM guidelines [21]. Similarly, in Pelotas, Brazil, we observed that ∼61% of females had insufficient weight gain. In contrast, our sample showed 46% of females gaining below and 15% above the IOM recommendations, whereas in MINA-Brazil, around 32% gained either below or above the recommendations [21].

Additionally, our ability to investigate the association between GWG >75th centile and health outcomes was limited by small sample sizes—only 23 females showed consistent GWG above the 75th centile, and only 5 females with underweight gained above this threshold at any point during pregnancy. Overall, only 7% of participants had GWG >75th centile at any point in pregnancy, which contributed to wider error bars and reduced statistical precision for this group.

As this was a secondary analysis, we were restricted to evaluating outcomes that were already collected. Some outcomes, such as pre-eclampsia (n = 59/3354), had small sample sizes, limiting our ability to assess associations with GWG parameters and generate precise estimates.

Another limitation is that the INTERBIO-21st Fetal Study did not include females with a BMI >35 kg/m^2^, limiting our ability to examine associations among individuals with severe obesity—a high-risk group for adverse outcomes. Additionally, only 2.4% of the sample (*n* = 80) comprised pregnant females >40 y, a group also at higher risk of complications. Finally, BMI categories were assigned based on first-trimester weight, as we lacked reliable prepregnancy weight data. This introduces the potential for BMI misclassification. These possible sources of selection bias can limit the generalizability of our findings to broader populations.

### Conclusion

Our study emphasizes the importance of GWG as a modifiable factor in pregnancy, highlighting the need for globally standardized guidelines. By mapping GWG trajectories against the IG standard, we provide a tool to monitor GWG within the <25th and >75th centile thresholds. This guideline offers a potential opportunity in minimizing the risk of adverse outcomes, particularly in females with normal weight. However, Further research is needed to extend these guidelines to females with underweight, overweight, and obesity to create a globally relevant GWG tool.

In conclusion, integrating gestational age- and BMI-specific GWG recommendations into global health policies is crucial for enhancing prenatal care and improving outcomes for mothers and children worldwide.

## Author contributions

The authors’ responsibilities were as follows – JV, SHK: responsible for conceiving the INTERGROWTH-21st Project; EOO: conceived the protocol for this analysis; NJ: performed the analysis with statistical oversight from LM and EOO and wrote the paper in collaboration with LM, JV, SHK, GO, LCI, ATP, and EOO; and LM, JV, SHK, GO, LCI, ATP, EOO: read the report, made suggestions about its content and approved the final version of the manuscript.

## Data availability

Data described in the manuscript, code book, and analytic code will be made available upon request.

## Funding

The INTERBIO-21st study was supported by the Bill & Melinda Gates Foundation under grant number 49038. The analyses of this work were funded by the Grant Challenges for Africa on Data Science Approaches to improve Maternal, Neonatal and Child Health in Africa. The funders were not involved in the analysis or writing of the study.

## Conflict of interest

The authors report no conflicts of interest.

## References

[1] Shenassa E.D., Kinsey C., Moser Jones M., Fahey J. (2017). Gestational weight gain: historical evolution of a contested health outcome. Obstet. Gynecol. Surv..

[2] Kominiarek M.A., Peaceman A.M. (2017). Gestational weight gain. Am. J. Obstet. Gynecol..

[3] Rasmussen K.M., Catalano P.M., Yaktine A.L. (2009). New guidelines for weight gain during pregnancy: what obstetrician/gynecologists should know. Curr. Opin. Obstet. Gynecol..

[4] Siega-Riz A.M., Bodnar L.M., Stotland N.E., Stang J. (2020). The current understanding of gestational weight gain among women with obesity and the need for future research. NAM Perspect.

[5] Muktabhant B., Lawrie T.A., Lumbiganon P., Laopaiboon M. (2015). Diet or exercise, or both, for preventing excessive weight gain in pregnancy. Cochrane Database Syst. Rev..

[6] Pugh S.J., Albert P.S., Kim S., Grobman W., Hinkle S.N., Newman R.B. (2017). Patterns of gestational weight gain and birthweight outcomes in the Eunice Kennedy Shriver National Institute of Child Health and Human Development Fetal Growth Studies–Singletons: a prospective study. Am. J. Obstet. Gynecol..

[7] Goldstein R.F., Abell S.K., Ranasinha S., Misso M.L., Boyle J.A., Harrison C.L. (2018). Gestational weight gain across continents and ethnicity: systematic review and meta-analysis of maternal and infant outcomes in more than one million women. BMC Med.

[8] Darling A.M., Wang D., Perumal N., Liu E., Wang M., Ahmed T. (2023). Risk factors for inadequate and excessive gestational weight gain in 25 low- and middle-income countries: an individual-level participant meta-analysis. PLOS Med.

[9] Perumal N., Wang D., Darling A.M., Liu E., Wang M., Ahmed T. (2023). Suboptimal gestational weight gain and neonatal outcomes in low and middle income countries: individual participant data meta-analysis. BMJ.

[10] Riddell C.A., Platt R.W., Bodnar L.M., Hutcheon J.A. (2017). Classifying gestational weight gain trajectories using the SITAR growth model. Paediatr. Perinat. Epidemiol..

[11] Cole T.J., Donaldson M.D.C., Ben-Shlomo Y. (2010). SITAR—a useful instrument for growth curve analysis. Int. J. Epidemiol..

[12] Cheikh Ismail L., Bishop D.C., Pang R., Ohuma E.O., Kac G., Abrams B. (2016). Gestational weight gain standards based on women enrolled in the Fetal Growth Longitudinal Study of the INTERGROWTH-21^st^ Project: a prospective longitudinal cohort study. BMJ.

[13] Villar J., Gunier R.B., Tshivuila-Matala C.O.O., Rauch S.A., Nosten F., Ochieng R. (2021). Fetal cranial growth trajectories are associated with growth and neurodevelopment at 2 years of age: INTERBIO-21st Fetal Study. Nat. Med..

[14] Ohuma E.O., Jabin N., Young M.F., Epie T., Martorell R., Peña-Rosas J.P. (2023). Association between maternal haemoglobin concentrations and maternal and neonatal outcomes: the prospective, observational, multinational, INTERBIO-21st fetal study. Lancet Haematol.

[15] Villar J., Ismail L.C., Victora C.G., Ohuma E.O., Bertino E., Altman D.G. (2014). International standards for newborn weight, length, and head circumference by gestational age and sex: the Newborn Cross-Sectional Study of the INTERGROWTH-21st project. Lancet.

[16] Villar J., Giuliani F., Fenton T.R., Ohuma E.O., Ismail L.C., Kennedy S.H. (2016). INTERGROWTH-21st very preterm size at birth reference charts. Lancet.

[17] Arora P., Tamber Aeri B. (2019). Gestational weight gain among healthy pregnant women from Asia in comparison with Institute of Medicine (IOM) guidelines-2009: a systematic review. J. Pregnancy..

[18] Villar J., Altman D.G., Purwar M., Noble J.A., Knight H.E., Ruyan P. (2013). The objectives, design and implementation of the INTERGROWTH-21^st^ project. BJOG.

[19] Hutcheon J.A., Bodnar L.M. (2018). Good practices for observational studies of maternal weight and weight gain in pregnancy. Paediatr. Perinat. Epidemiol..

[20] Gilmore L.A., Redman L.M. (2015). Weight gain in pregnancy and application of the 2009 IOM guidelines: toward a uniform approach. Obesity (Silver Spring).

[21] Mosquera P.S., Malta M.B., De Araújo Damasceno A.A., Neves P.A.R., Matijasevich A., Cardoso M.A. (2022). Associations of gestational weight gain with perinatal outcomes in Western Brazilian Amazon, Matern. Child Health J.

[22] Thiruvengadam R., Desiraju B.K., Natchu U.C.M., Wadhwa N., Sachdeva K., Misra S. (2022). Gestational weight gain trajectories in GARBH–Ini pregnancy cohort in North India and a comparative analysis with global references. Eur. J. Clin. Nutr..

[23] Jin C., Lin L., Han N., Zhao Z., Liu Z., Luo S. (2019). Excessive gestational weight gain and the risk of gestational diabetes: comparison of INTERGROWTH-21st standards, IOM recommendations and a local reference. Diabetes Res. Clin. Pract..

[24] Ouedraogo D.S., Compaore E.W.R., Ouedraogo O., Dicko M.H. (2024). Associated factors of dietary diversity among schoolchildren in Plateau Central region of Burkina Faso: a cross-sectional study. BMC Nutr.

[25] Voerman E., Santos S., Inskip H., Amiano P., Barros H., LifeCycle Project-Maternal Obesity and Childhood Outcomes Study Group (2019). Association of gestational weight gain with adverse maternal and infant outcomes. JAMA.

[26] Bodnar L.M., Johansson K., Himes K.P., Khodyakov D., Abrams B., Parisi S.M. (2024). Do current pregnancy weight gain guidelines balance risks of adverse maternal and child health in a United States cohort?. Am. J. Clin. Nutr..

[27] Liu X., Wang H., Yang L., Zhao M., Magnussen C.G., Xi B. (2022). Associations between gestational weight gain and adverse birth outcomes: a population-based retrospective cohort study of 9 million mother-infant Pairs. Front. Nutr..

[28] Macdonald-Wallis C., Tilling K., Fraser A., Nelson S.M., Lawlor D.A. (2013). Gestational weight gain as a risk factor for hypertensive disorders of pregnancy. Am. J. Obstet. Gynecol..

[29] Goldstein R.F., Abell S.K., Ranasinha S., Misso M., Boyle J.A., Black M.H. (2017). Association of gestational weight gain with maternal and infant outcomes: a systematic review and meta-analysis. JAMA.

[30] Bodnar L.M., Johansson K., Himes K.P., Khodyakov D., Abrams B., Parisi S.M. (2024). Gestational weight gain below recommendations and adverse maternal and child health outcomes for pregnancies with overweight or obesity: a United States cohort study. Am. J. Clin. Nutr..

[31] (1995). Physical status: the use and interpretation of anthropometry, Report of a WHO Expert Committee. World Health Organ. Tech. Rep. Ser..

[32] First global call for data on gestational weight gain. Accessed March 26, 2024. Available from: https://www.who.int/news-room/articles-detail/first-global-call-for-data-on-gestational-weight-gain.

