# Supplementary material for: Association between gestational age-specific weight gain in pregnancy and risk of adverse perinatal outcomes: a secondary analysis of the INTERBIO-21st Fetal Study
Source: Am J Clin Nutr. 2025 Apr 16;121(6):1304–14. doi: 10.1016/j.ajcnut.2025.04.012 (PMC12226757; doi:10.1016/j.ajcnut.2025.04.012)
Supplement: Multimedia component 1 [file mmc1.docx]

Contents

[**Supplementary Table S1**: Variables adjusted in multivariable regression analyses 3](#_Toc194072320)

[**Supplementary Table S2**: Frequency of gestational age specific gestational weight gain (GWG) INTERGROWTH-21^st^ (IG) standards and maternal BMI specific Institute of Medicine (IOM) GWG standard among 3354 women by early pregnancy body mass index (BMI) categories. 4](#_Toc194072321)

[**Supplementary Table S3**: Baseline socio-demographic characteristics of participants in the INTERBIO-21^st^ Fetal Study (N=3354) 5](#_Toc194072322)

[**Supplementary Table S4**: Maternal gestational weight gain and perinatal outcomes by study sites in the INTERBIO-21^st^ Fetal Study (N= 3354) 6](#_Toc194072323)

[**Supplementary Figure S1**: Odds ratios (ORs) with 95% confidence intervals (CIs) for the association between perinatal health outcomes and gestational age-specific weight gain below the 25^th^ centile at any point of pregnancy in reference to women gaining consistently between 25^th^ and 75^th^ centile of the INTERGROWTH-21^st^ (IG) standard, in the overall sample and subgroups of women with different levels of body mass index (BMI). 7](#_Toc194072324)

[**Supplementary Figure S2a:** Odds ratios (ORs) with 95% confidence intervals (CIs) for the association between perinatal health outcomes (gestational diabetes mellitus [GDM], small for gestational age [SGA], preterm birth [PTB], low birthweight [LBW], birth length <10^th^ centile, and birth head circumference <10^th^ centile) and gestational weight gain below the IOM gestational weight gain standard, in the overall sample and across maternal body mass index (BMI) categories. 8](#_Toc194072325)

[**Supplementary Figure S2b**: Odds ratios (ORs) with 95% confidence intervals (CIs) for the association between neonatal health outcomes (pregnancy induced hypertension [PIH], gestational diabetes mellitus [GDM], emergency cesarean section [C-section], large for gestational age [LGA], macrosomia, birth length >90^th^ centile, and birth head circumference>90^th^ centile) and gestational weight gain above the IOM gestational weight gain standard, in the overall sample and across maternal body mass index (BMI) categories. 9](#_Toc194072326)

[**Supplementary Figure S3:** Odds Ratio of gestational diabetes mellitus (GDM) with 95% confidence intervals (CI) for GWG centiles: <3^rd^, <5^th^, <10^th^, and <25^th^ compared to GWG between the 25^th^ and 75^th^ centiles from multivariable regression model adjusted for possible confounding factors: maternal age, maternal BMI, maternal education, smoking habit, and previous history of gestational diabetes. 10](#_Toc194072327)

[**Supplementary Figure S4:** Odds Ratio of preterm birth, PTB (birth before 37 weeks of pregnancy) with 95% confidence intervals (CI) for GWG centiles: <3^rd^, <5^th^, <10^th^, and <25^th^ compared to GWG between the 25^th^ and 75^th^ centiles from multivariable regression model adjusted for potential confounders: maternal age, maternal BMI, maternal education, smoking habit, alcohol consumption, nulliparity, gestational diabetes, pregnancy induced hypertension, history of hypertension, diabetes, and infant sex. 11](#_Toc194072328)

[**Supplementary Figure S5:** Odds Ratio of low birth weight, LBW (birthweight less than 2500g) with 95% confidence intervals (CI) for GWG centiles: <3^rd^, <5^th^, <10^th^, and <25^th^ compared to GWG between the 25^th^ and 75^th^ centiles from multivariable regression model adjusted for potential confounders: maternal age, maternal BMI, maternal education, smoking habit, alcohol consumption, nulliparity, gestational diabetes, pregnancy induced hypertension, history of hypertension, diabetes, and infant sex. 12](#_Toc194072329)

[**Supplementary Figure S6:** Odds Ratio of small for gestational age, SGA (birthweight below 10^th^ centile) with 95% confidence intervals (CI) for GWG centiles: <3^rd^, <5^th^, <10^th^, and <25^th^, compared to GWG between the 25^th^ and 75^th^ centiles from multivariable regression model adjusted for potential confounders: maternal age, maternal BMI, maternal education, smoking habit, alcohol consumption, nulliparity, gestational diabetes, pregnancy induced hypertension, history of hypertension, and infant sex. 13](#_Toc194072330)

[**Supplementary Figure S7:** Odds Ratio of birth length less than 10^th^ centile with 95% confidence intervals (CI) for GWG centiles: <3^rd^, <5^th^, <10^th^, and <25^th^ compared to GWG between the 25^th^ and 75^th^ centiles from multivariable regression model adjusted for potential confounders: maternal age, maternal BMI, maternal education, smoking habit, alcohol consumption, nulliparity, gestational diabetes, pregnancy induced hypertension, history of hypertension, diabetes, and infant sex. 14](#_Toc194072331)

[**Supplementary Figure S8:** Odds Ratio of birth head circumference less than 10^th^ centile with 95% confidence intervals (CI) for GWG centiles: <3^rd^, <5^th^, <10^th^, and <25^th^ compared to GWG between the 25^th^ and 75^th^ centiles from multivariable regression model adjusted for potential confounders: maternal age, maternal BMI, maternal education, smoking habit, alcohol consumption, nulliparity, gestational diabetes, pregnancy induced hypertension, history of hypertension, diabetes, and infant sex. 15](#_Toc194072332)

[**Supplementary Table S5**: Maternal and neonatal health outcomes by gestational weight gain categories in a cohort of 3354 women 16](#_Toc194072333)

[**Supplementary Figure S9**: Adjusted odds ratios (ORs) with 95% confidence interval (CIs) of maternal and neonatal health outcomes for women gaining consistently <25^th^ centile GWG throughout pregnancy. These odds ratios were performed in the overall sample and in high- (UK) and low-income countries (Brazil, Kenya, Pakistan, South Africa and Thailand). 17](#_Toc194072334)

[**Supplementary Figure S10**: Adjusted odds ratio with 95% confidence interval of maternal and neonatal health outcomes for women gaining consistently <25^th^ centile GWG throughout the pregnancy. The odds ratio were performed excluding each site at a time. 18](#_Toc194072335)

[**Supplementary Figure S11**: Adjusted odds ratio with 95% confidence interval of maternal and neonatal health outcomes for women gaining consistently <25^th^ centile GWG throughout the pregnancy. The odds ratio were performed in the overall sample and in study sites that contributed to INTERGROWTH-21^st^ (IG) study (UK, Kenya, and Brazil) and sites that contributed only to the INTERBIO-21^st^ study (Pakistan, South Africa, and Thailand). 19](#_Toc194072336)

[**Supplementary Table S6**: Adjusted odds ration with 95% confidence interval (CI) of maternal and neonatal health outcomes for gestational weight gain centiles by trimester for 3354 women 20](#_Toc194072337)

# **Supplementary Table S1**: Variables adjusted in multivariable regression analyses

| **Outcomes** | **Adjusted variables** |
| --- | --- |
| **Maternal outcomes** |  |
| Pregnancy induced Hypertension (PIH) | Maternal age, maternal education, maternal BMI, smoking habit, alcohol consumption, and countries |
| Gestational diabetes Mellitus (GDM) | Maternal age, maternal education, maternal BMI, smoking habit, alcohol consumption, and countries |
| Emergency cesarean delivery | Maternal age, maternal education, maternal BMI, smoking habit, alcohol consumption, nulliparity, gestational diabetes, pregnancy induced hypertension, previous history of hypertension, and countries |
| **Neonatal outcomes** |  |
| Preterm birth (PTB) | Maternal age, maternal education, maternal BMI, smoking habit, alcohol consumption, nulliparity, gestational diabetes, pregnancy induced hypertension, previous history of hypertension, diabetes, infant sex, and countries |
| Low birth weight (LBW) | Maternal age, maternal education, maternal BMI, smoking habit, alcohol consumption, nulliparity, gestational diabetes, pregnancy induced hypertension, previous history of hypertension, diabetes, infant sex, and countries |
| Macrosomia | Maternal age, maternal education, maternal BMI, smoking habit, alcohol consumption, nulliparity, gestational diabetes, pregnancy induced hypertension, previous history of hypertension, diabetes, infant sex, and countries |
| Small for gestational age (SGA) | Maternal age, maternal education, maternal BMI, smoking habit, alcohol consumption, nulliparity, gestational diabetes, pregnancy induced hypertension, previous history of hypertension and countries |
| Large for gestational age (LGA) | Maternal age, maternal education, maternal BMI, smoking habit, alcohol consumption, nulliparity, gestational diabetes, diabetes and countries |
| Birth Length <10^th^ centile | Maternal age, maternal education, maternal BMI, smoking habit, alcohol consumption, nulliparity, gestational diabetes, pregnancy induced hypertension, previous history of hypertension, diabetes, and countries |
| Birth Length>90^th^ centile | Maternal age, maternal education, maternal BMI, smoking habit, alcohol consumption, nulliparity, gestational diabetes, pregnancy induced hypertension, previous history of hypertension, diabetes, and countries |
| Birth Head circumference <10^th^ centile | Maternal age, maternal education, maternal BMI, smoking habit, alcohol consumption, nulliparity, gestational diabetes, pregnancy induced hypertension, previous history of hypertension, diabetes, and countries |
| Birth Head circumference >90^th^ centile | Maternal age, maternal education, maternal BMI, smoking habit, alcohol consumption, nulliparity, gestational diabetes, pregnancy induced hypertension, previous history of hypertension, diabetes, and countries |

# **Supplementary Table S2**: Frequency of gestational age specific gestational weight gain (GWG) INTERGROWTH-21^st^ (IG) standards and maternal BMI specific Institute of Medicine (IOM) GWG standard among 3354 women by early pregnancy body mass index (BMI) categories.

|  | **Underweight** | **Normal weight** | **Overweight** | **Obese** | **Overall** |
| --- | --- | --- | --- | --- | --- |
|  | **(N=170)** | **(N=1686)** | **(N=1026)** | **(N=471)** | **(N=3354)** |
| **Gestational age specific IG GWG standards** |  |  |  |  |  |
| GWG <3^rd^ centile throughout pregnancy | 18 (10.6%) | 125 (7.4%) | 120 (11.7%) | 103 (21.9%) | 367 (10.9%) |
| GWG <5^th^ centile throughout pregnancy | 27 (15.9%) | 199 (11.8%) | 180 (17.5%) | 138 (29.3%) | 545 (16.2%) |
| GWG <10^th^ centile throughout pregnancy | 52 (30.6%) | 373 (22.1%) | 302 (29.4%) | 198 (42.0%) | 926 (27.6%) |
| GWG <25^th^ centile throughout pregnancy | 94 (55.3%) | 802 (47.6%) | 558 (54.4%) | 312 (66.2%) | 1767 (52.7%) |
| GWG >75^th^ centile throughout pregnancy | 1 (0.6%) | 6 (0.4%) | 10 (1.0%) | 6 (1.3%) | 23 (0.7%) |
| GWG >90^th^ centile throughout pregnancy | 0 (0%) | 3 (0.2%) | 5 (0.5%) | 1 (0.2%) | 9 (0.3%) |
| GWG <25th centile or >75th centile throughout the pregnancy | 95 (55.9%) | 808 (47.9%) | 568 (55.4%) | 318 (67.5%) | 1790 (53.4%) |
| GWG <25^th^ centile at any point of pregnancy | 146 (85.9%) | 1375 (81.6%) | 864 (84.2%) | 415 (88.1%) | 2801 (83.5%) |
| GWG >75^th^ centile at any point of pregnancy | 5 (2.9%) | 129 (7.7%) | 79 (7.7%) | 30 (6.4%) | 243 (7.2%) |
| GWG <25^th^ centile or >75^th^ centile at any point of pregnancy | 150 (88.2%) | 1470 (87.2%) | 924 (90.1%) | 439 (93.2%) | 2984 (89.0%) |
| GWG between 25^th^ centile and 75^th^ centile throughout pregnancy | 20 (11.8%) | 216 (12.8%) | 102 (9.9%) | 32 (6.8%) | 370 (11.0%) |
| **BMI specific IOM GWG standards** |  |  |  |  |  |
| GWG less than or above IOM recommended range | 156 (91.8%) | 1364 (80.9%) | 634 (61.8%) | 293 (62.2%) | 2447 (73.0%) |
| GWG less than IOM lower limit | 155 (91.2%) | 1309 (77.6%) | 441 (43.0%) | 174 (36.9%) | 2079 (62.0%) |
| GWG more than IOM upper limit | 1 (0.6%) | 55 (3.3%) | 193 (18.8%) | 119 (25.3%) | 368 (11.0%) |
| GWG between IOM recommended range | 14 (8.2%) | 322 (19.1%) | 392 (38.2%) | 178 (37.8%) | 906 (27.0%) |

IG standards are gestational age specific. IOM recommended range: underweight (12.5-18 kg), normal weight (11.5-16 kg), overweight (7-11.5 kg), and obese (5-9 kg) during pregnancy. According to the IOM guidelines, GWG below the IOM standard is defined as follows: underweight women (<18.5 kg/m²) gaining less than 12.5 kg, normal weight women (18.5-24.9 kg/m²) gaining less than 11.5 kg, overweight women (25-29.9 kg/m²) gaining less than 7 kg, and women with obesity (≥30 kg/m²) gaining less than 5 kg during pregnancy. According to the IOM guidelines, GWG above the IOM standard is defined as follows: underweight women (<18.5 kg/m²) gaining more than 18 kg, normal weight women (18.5-24.9 kg/m²) gaining more than 16 kg, overweight women (25-29.9 kg/m²) gaining more than 11.5 kg, and women with obesity (≥30 kg/m²) gaining more than 9 kg during pregnancy.

# **Supplementary Table S3**: Baseline socio-demographic characteristics of participants in the INTERBIO-21^st^ Fetal Study (N=3354)

| **Characteristic** | **Pelotas**  **(Brazil)**  **N = 400** | **Nairobi**  **(Kenya)**  **N = 586** | **Karachi (Pakistan)**  **N = 535** | **Soweto**  **(South Africa)**  **N = 607** | **Mae Sot (Thailand)**  **N = 566** | **Oxford**  **(UK)**  **N = 660** |
| --- | --- | --- | --- | --- | --- | --- |
| **Mother’s age (years),** Median (IQR) | 29 (25, 33) | 31 (28, 33) | 30 (27, 33) | 31 (27, 36) | 25 (21, 30) | 32 (28, 34) |
| **Father’s age (years)**, Median (IQR) | 30 (26, 34) | 33 (30, 36) | 33 (30, 37) | 35 (30, 39) | 28 (24, 34) | 33 (29, 37) |
| **Marital status, n (%)** |  |  |  |  |  |  |
| Married/Cohabiting | 364 (91.0%) | 531 (90.6%) | 533 (99.6%) | 241 (39.7%) | 560 (99.0%) | 628 (95.2%) |
| Single | 34 (8.6%) | 55 (9.4%) | 1 (0.2%) | 364 (60.0%) | 4 (0.7%) | 30 (4.5%) |
| Separated/Divorced | 1 (0.3%) | 0 (0.0%) | 1 (0.2%) | 2 (0.3%) | 0 (0.0%) | 2 (0.3%) |
| Widowed | 1 (0.3%) | 0 (0.0%) | 0 (0.0%) | 0 (0.0%) | 2 (0.3%) | 0 (0.0%) |
| **Mother’s education level, n (%)** |  |  |  |  |  |  |
| No school attended | 0 (0.0%) | 0 (0.0%) | 4 (0.7%) | 1 (0.2%) | 148 (26.1%) | 0 (0.0%) |
| Primary | 63 (15.8%) | 2 (0.3%) | 18 (3.4%) | 18 (3.0%) | 236 (41.7%) | 0 (0.0%) |
| Professional/technical training | 25 (6.3%) | 191 (32.6%) | 23 (4.3%) | 100 (16.5%) | 5 (0.9%) | 119 (18.0%) |
| Secondary | 170 (42.5%) | 5 (0.9%) | 91 (17.0%) | 444 (73.1%) | 167 (29.5%) | 237 (35.9%) |
| University | 142 (35.5%) | 388 (66.2%) | 399 (74.6%) | 44 (7.2%) | 10 (1.8%) | 304 (46.1%) |
| **Mother’s occupation, n (%)** |  |  |  |  |  |  |
| Clerical support, service or sales | 120 (30.0%) | 62 (10.6%) | 0 (0.0%) | 82 (13.5%) | 5 (0.9%) | 203 (30.8%) |
| Housework | 98 (24.5%) | 21 (3.6%) | 347 (64.9%) | 66 (10.9%) | 360 (63.6%) | 105 (15.9%) |
| Managerial/professional/technical | 81 (20.3%) | 452 (77.1%) | 143 (26.7%) | 18 (3.0%) | 14 (2.5%) | 304 (46.1%) |
| Skilled manual work | 23 (5.8%) | 12 (2.0%) | 34 (6.4%) | 51 (8.4%) | 42 (7.4%) | 6 (0.9%) |
| Unskilled manual work | 6 (1.5%) | 0 (0.0%) | 2 (0.4%) | 65 (10.7%) | 134 (23.7%) | 20 (3.0%) |
| Student | 22 (5.5%) | 27 (4.6%) | 6 (1.1%) | 22 (3.6%) | 0 (0.0%) | 21 (3.2%) |
| Other | 49 (12.3%) | 12 (2.0%) | 3 (0.6%) | 303 (49.9%) | 11 (1.9%) | 1 (0.2%) |
| **Mother’s height (cm),** Median (IQR) | 162 (158, 166) | 162 (158, 166) | 158 (154, 162) | 159 (155, 163) | 152 (149, 155) | 165 (161, 170) |
| **Mother’s weight (kg),** Median (IQR) | 66 (59, 76) | 66 (58, 75) | 63 (55, 70) | 66 (59, 76) | 47 (43, 52) | 65 (58, 75) |
| **Mother’s BMI (kg/m^2^),** Median (IQR) | 25 (22, 28) | 25 (22, 28) | 25 (22, 28) | 27 (23, 30) | 21 (19, 23) | 24 (21, 27) |
| **Mother’s BMI status, n (%)** |  |  |  |  |  |  |
| Underweight (BMI < 18.5 kg/m²) | 7 (1.8%) | 12 (2.1%) | 25 (4.7%) | 12 (2.0%) | 97 (17.2%) | 17 (2.6%) |
| Normal weight (BMI 18.5–24.9 kg/m²) | 184 (46.0%) | 254 (43.4%) | 235 (43.9%) | 212 (34.9%) | 415 (73.3%) | 386 (58.5%) |
| Overweight (BMI 25–29.9 kg/m²) | 148 (37.0%) | 225 (38.5%) | 194 (36.3%) | 232 (38.2%) | 50 (8.8%) | 177 (26.8%) |
| Obese (BMI ≥ 30 kg/m²) | 61 (15.2%) | 94 (16.0%) | 81 (15.1%) | 151 (24.9%) | 4 (0.7%) | 80 (12.1%) |
| **Nulliparity, n (%)** | 234 (58.5%) | 226 (38.6%) | 130 (24.3%) | 38 (6.3%) | 170 (30.0%) | 194 (29.4%) |
| **Smoking, n (%)** | 30 (7.5%) | 0 (0.0%) | 1 (0.2%) | 35 (5.9%) | 50 (9.3%) | 53 (8.1%) |
| **Alcohol consumption, n (%)** | 15 (3.8%) | 2 (0.3%) | 0 (0.0%) | 48 (8.1%) | 1 (0.2%) | 14 (2.1%) |

# **Supplementary Table S4**: Maternal gestational weight gain and perinatal outcomes by study sites in the INTERBIO-21^st^ Fetal Study (N= 3354)

| Characteristic | **Pelotas**  **(Brazil)**  **N = 400** | **Nairobi**  **(Kenya)**  **N = 586** | **Karachi**  **(Pakistan)**  **N = 535** | **Soweto**  **(South Africa)**  **N = 607** | **Mae Sot**  **(Thailand)**  **N = 566** | **Oxford**  **(UK)**  **N = 660** |
| --- | --- | --- | --- | --- | --- | --- |
| **Total gestational weight gain (kg), Median (IQR)** | 8.9 (6.8, 11.7) | 8.1 (5.9, 10.6) | 6.3 (4.1, 8.8) | 7.0 (4.2, 9.8) | 7.7 (5.5, 10.0) | 10.1 (7.4, 12.7) |
| **GWG <25th centile at any point of pregnancy** | 170 (42.5%) | 321 (54.8%) | 353 (66.0%) | 355 (58.5%) | 336 (59.4%) | 232 (35.2%) |
| **GWG >75th centile at any point of pregnancy** | 33 (8.3%) | 31 (5.3%) | 28 (5.2%) | 42 (6.9%) | 28 (4.9%) | 81 (12% |
| **GWG less than IOM lower limit** | 184 (46.0%) | 341 (58.0%) | 394 (74.0%) | 362 (60.0%) | 470 (83.0%) | 328 (50.0%) |
| **GWG above IOM upper limit** | 60 (15.0%) | 72 (12.0%) | 22 (4.1%) | 67 (11.0%) | 14 (2.5%) | 133 (20.0%) |
| **Gestational age at delivery (weeks), Median (IQR)** | 38.9  (38.0, 39.6) | 40.0  (39.0, 40.7) | 38.0  (37.1, 39.0) | 38.9  (37.7, 39.9) | 39.7  (38.9, 40.3) | 39.9  (38.7, 40.7) |
| **Pre-eclampsia, n (%)** | 13 (4.1%) | 9 (3.0%) | 17 (4.3%) | 8 (2.2%) | 3 (6.3%) | 9 (3.6%) |
| **Pregnancy induced hypertension, n (%)** | 20 (5.1%) | 8 (1.4%) | 32 (6.2%) | 23 (4.0%) | 20 (3.8%) | 41 (6.4%) |
| **Gestational diabetes, n (%)** | 29 (7.3%) | 13 (2.2%) | 127 (24.0%) | 7 (1.2%) | 7 (1.3%) | 21 (3.2%) |
| **Mode of Delivery, n (%)** |  |  |  |  |  |  |
| Assisted breech | 0 (0.0%) | 0 (0.0%) | 0 (0.0%) | 0 (0.0%) | 3 (0.6%) | 1 (0.2%) |
| Caesarean section | 307 (77.0%) | 219 (37.4%) | 309 (59.0%) | 336 (57.0%) | 16 (3.0%) | 119 (18.0%) |
| Vaginal assisted | 3 (0.8%) | 15 (2.6%) | 17 (3.3%) | 2 (0.3%) | 15 (2.4%) | 102 (17.8%) |
| Vaginal spontaneous | 89 (22.2%) | 349 (60.0%) | 197 (37.7%) | 253 (42.7%) | 501 (94.0%) | 431 (66.0%) |
| **Emergency caesarean delivery, n (%)** | 162 (41.0%) | 132 (23.0%) | 140 (26.0%) | 195 (32.0%) | 13 (2.3%) | 72 (11.0%) |
| **Caesarean delivery, n (%)** | 307 (77.0%) | 219 (37.0%) | 309 (58.0%) | 336 (55.0%) | 16 (2.8%) | 119 (18.0%) |
| **Preterm birth, n (%)** | 50 (13.0%) | 33 (5.7%) | 107 (20.0%) | 115 (19.0%) | 34 (6.4%) | 56 (8.6%) |
| **Newborn sex: Male, n (%)** | 218 (55.0%) | 305 (52.0%) | 253 (48.0%) | 305 (52.0%) | 274 (51.0%) | 343 (53.0%) |
| **Birthweight (grams)^1^, Median (IQR)** | 3,150  (2,818, 3,485) | 3,265  (2,990, 3,560) | 2,900  (2,600, 3,194) | 2,990  (2,600, 3,260) | 2,960  (2,726, 3,218) | 3,320  (2,985, 3,655) |
| **Low Birth weight, n (%)** | 37 (9.3%) | 30 (5.2%) | 87 (17.0%) | 113 (20.0%) | 69 (13.0%) | 53 (8.1%) |
| **Macrosomia, n (%)** | 10 (2.5%) | 27 (4.7%) | 0 (0.0%) | 16 (2.8%) | 4 (0.8%) | 61 (9.3%) |
| **Small for gestational age (SGA)^2^, n (%)** | 36 (9.0%) | 63 (11.0%) | 65 (13.0%) | 128 (22.0%) | 125 (23.0%) | 49 (7.5%) |
| **Large for gestational age (LGA)^2^, n (%)** | 30 (7.5%) | 45 (7.8%) | 11 (2.1%) | 38 (6.5%) | 10 (1.9%) | 90 (14.0%) |
| **Birth length (cm)^1^, Median (IQR)** | 48.5  (47.2, 49.8) | 49.5  (48.3, 50.3) | 48.4  (46.8, 49.5) | 48.3  (46.4, 50.2) | 48.4  (47.2, 49.6) | 49.3  (47.7, 50.5) |
| **Birth length below 10th centile^2^, n (%)** | 41 (10.0%) | 45 (8.1%) | 45 (8.9%) | 121 (22.0%) | 115 (22.0%) | 65 (10.0%) |
| **Birth length above 90th centile^2^, n (%)** | 28 (7.0%) | 44 (7.9%) | 46 (9.1%) | 91 (16.0%) | 21 (4.0%) | 66 (10.0%) |
| **Birth head circumference (cm)^1^, Median (IQR)** | 33.8  (33.1, 34.8) | 34.5  (33.8, 35.3) | 33.4  (32.4, 34.1) | 34.0  (33.0, 35.0) | 33.0  (32.2, 33.8) | 34.3  (33.3, 35.1) |
| **Birth head circumference below 10th centile^2^, n (%)** | 14 (3.5%) | 21 (3.8%) | 35 (6.8%) | 38 (6.8%) | 158 (30.0%) | 34 (5.3%) |
| **Birth head circumference above 90th centile^2^, n (%)** | 52 (13.0%) | 95 (17.0%) | 35 (6.8%) | 125 (22.0%) | 9 (1.7%) | 1. 15.0%) |

1. Birthweight was missing for 112 (3.3%), birth length was missing for 168 (5.0%) and birth head circumference was missing for 155 (4.6%), mode of delivery information was missing for 42 women.

2. SGA, LGA, length<10th centile, length>90th centile, head circumference<10th centile, head circumference>90th centile were defined respectively as <10th and >90th centiles of the INTERGROWTH-21st Newborn Size Standard and Very Preterm Size at Birth Reference^29, 30^

**Supplementary Figure S1**: Odds ratios (ORs) with 95% confidence intervals (CIs) for the association between perinatal health outcomes and gestational age-specific weight gain below the 25^th^ centile at any point of pregnancy in reference to women gaining consistently between 25^th^ and 75^th^ centile of the INTERGROWTH-21^st^ (IG) standard, in the overall sample and subgroups of women with different levels of body mass index (BMI).


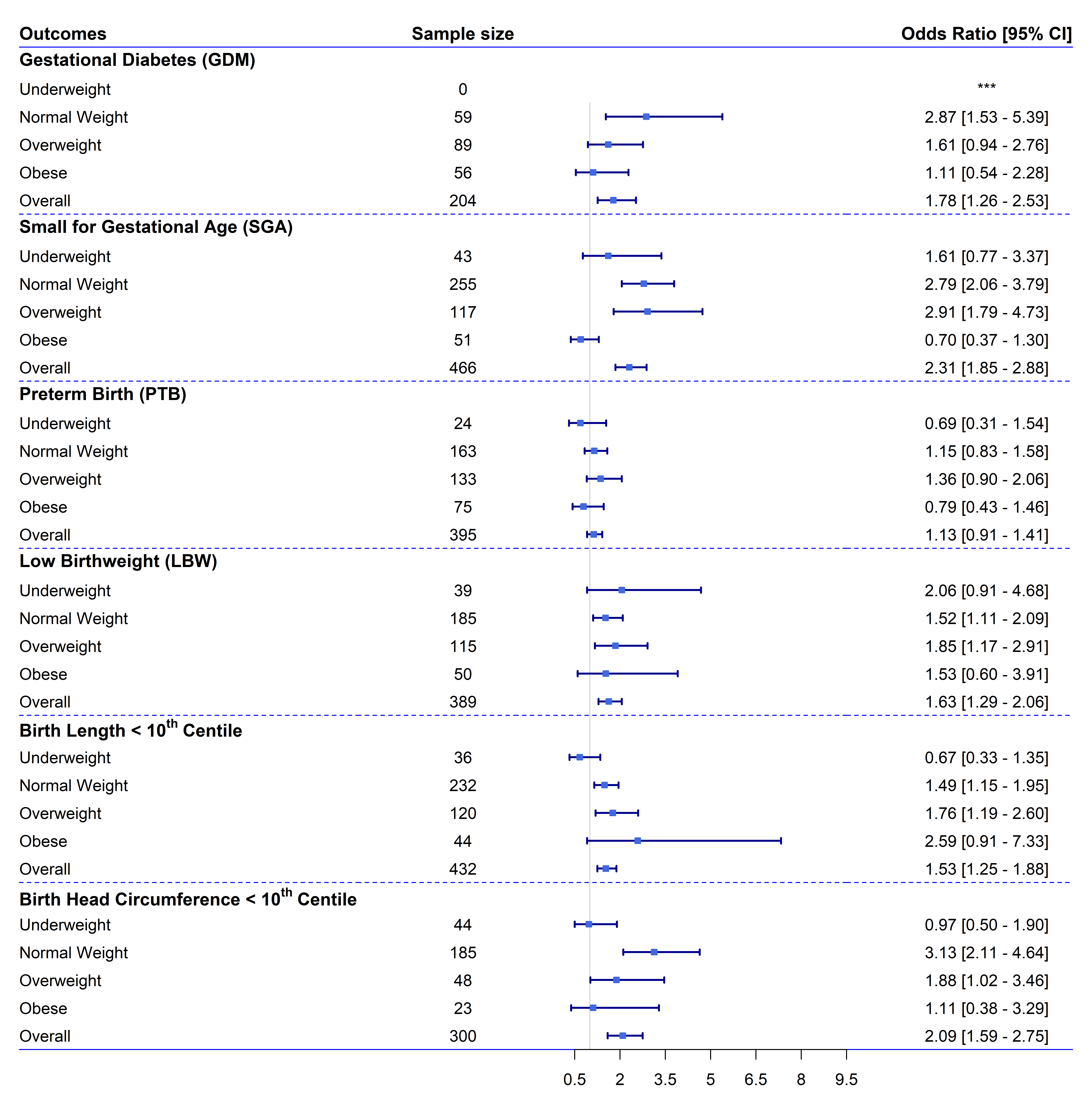


The odds ratios (ORs) and 95% confidence intervals (CIs) are shown for the association between gestational weight gain (GWG) below the 25^th^ centile of the gestational age-specific INTERGROWTH-21^st^ (IG) standard at any point of pregnancy (n=2801) and adverse perinatal health outcomes. These associations were analyzed using multivariable regression models adjusted for possible covariates. The associations are shown across early-pregnancy BMI categories: normal weight (n=1686), overweight (n=1026), and obese (n=471). The association could not be shown for underweight women due to the small sample size. The frequency of women gaining GWG < 25^th^ centile IG standard continuously throughout the pregnancy was 146 for underweight, 1375 for normal weight, 864 for overweight, and 415 for obese women. The reference group includes women gaining gestational weight between the 25^th^ and 75^th^ centiles of the IG standard during pregnancy (overall, n=370; underweight, n=20; normal weight, n=216; overweight, n=102; obese, n=32). For each outcome, the sample size represents the total number of women who experienced the outcome in each BMI category and in the overall sample.

# **Supplementary Figure S2a:** Odds ratios (ORs) with 95% confidence intervals (CIs) for the association between perinatal health outcomes (gestational diabetes mellitus [GDM], small for gestational age [SGA], preterm birth [PTB], low birthweight [LBW], birth length <10^th^ centile, and birth head circumference <10^th^ centile) and gestational weight gain below the IOM gestational weight gain standard, in the overall sample and across maternal body mass index (BMI) categories.


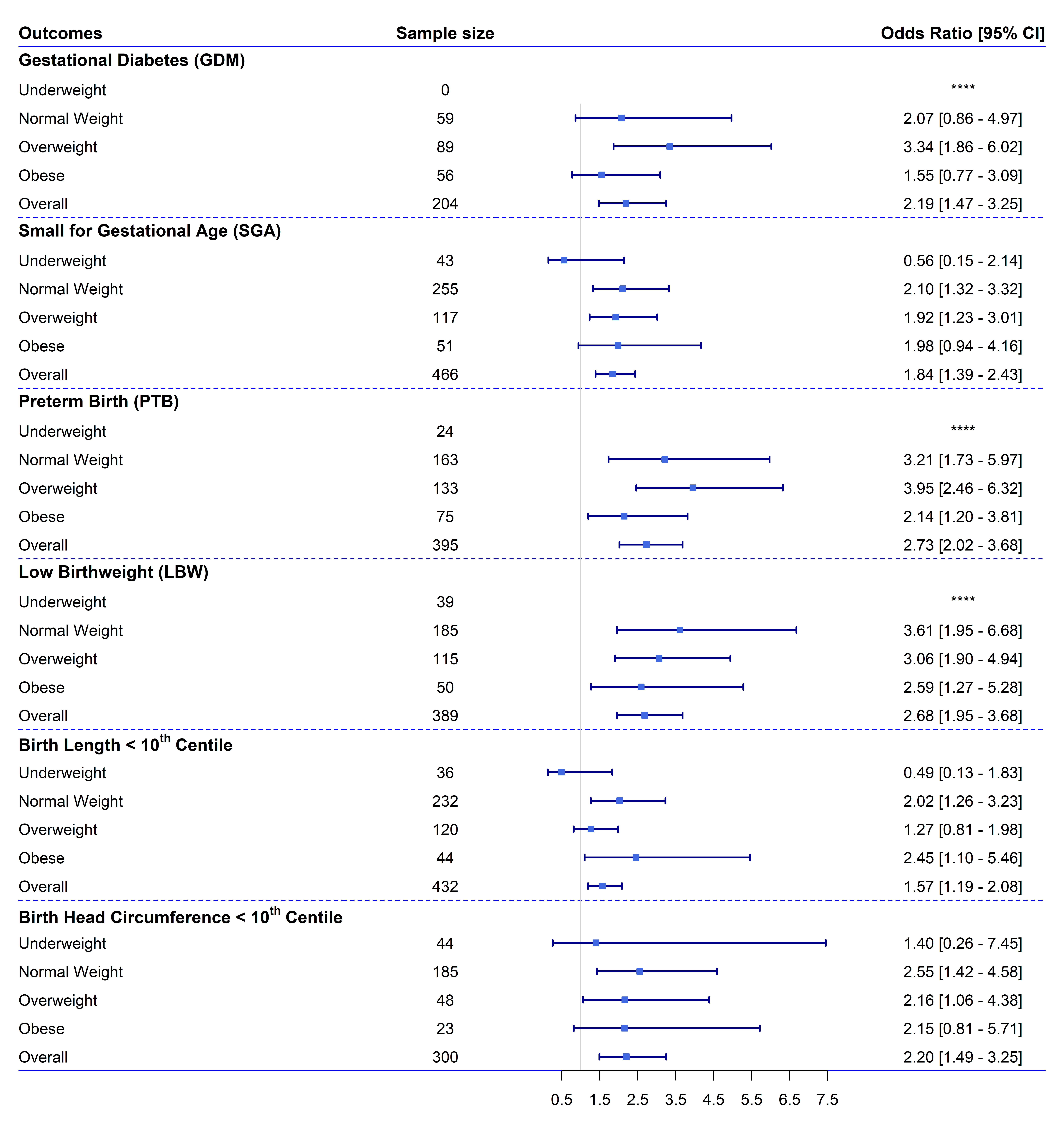


The odds ratios (ORs) and 95% confidence intervals (CIs) are shown for the association between gestational weight gain (GWG) below the maternal body mass index (BMI)-specific IOM standard during pregnancy (n=2079) and adverse maternal health outcomes. According to the IOM guidelines, GWG below the IOM standard is defined as follows: underweight women (<18.5 kg/m²) gaining less than 12.5 kg, normal weight women (18.5-24.9 kg/m²) gaining less than 11.5 kg, overweight women (25-29.9 kg/m²) gaining less than 7 kg, and women with obesity (≥30 kg/m²) gaining less than 5 kg during pregnancy. These associations were analyzed using multivariable regression models adjusted for possible covariates. The associations are shown across early-pregnancy BMI categories: underweight (n=170), normal weight (n=1686), overweight (n=1026), and obese (n=471). The prevalence of women gaining below the IOM standard was 1309 for normal weight, 441 for overweight, and 174 for obese women. The reference group includes women gaining gestational weight within the IOM recommended range: underweight (12.5-18 kg), normal weight (11.5-16 kg), overweight (7-11.5 kg), and obese (5-9 kg) during pregnancy. Overall, 906 women gained IOM recommended weight (underweight, n=14, normal weight, n=322; overweight, n=392; and obese, n=178). For each outcome, the sample size represents the total number of women who experienced the outcome in each BMI category and in the overall sample.

***prevalence of gestational diabetes was 0 among underweight women. Also, prevalence of preterm brith was 0 among the reference group, women gainng between the IOM standard

# **Supplementary Figure S2b**: Odds ratios (ORs) with 95% confidence intervals (CIs) for the association between neonatal health outcomes (pregnancy induced hypertension [PIH], gestational diabetes mellitus [GDM], emergency cesarean section [C-section], large for gestational age [LGA], macrosomia, birth length >90^th^ centile, and birth head circumference>90^th^ centile) and gestational weight gain above the IOM gestational weight gain standard, in the overall sample and across maternal body mass index (BMI) categories.


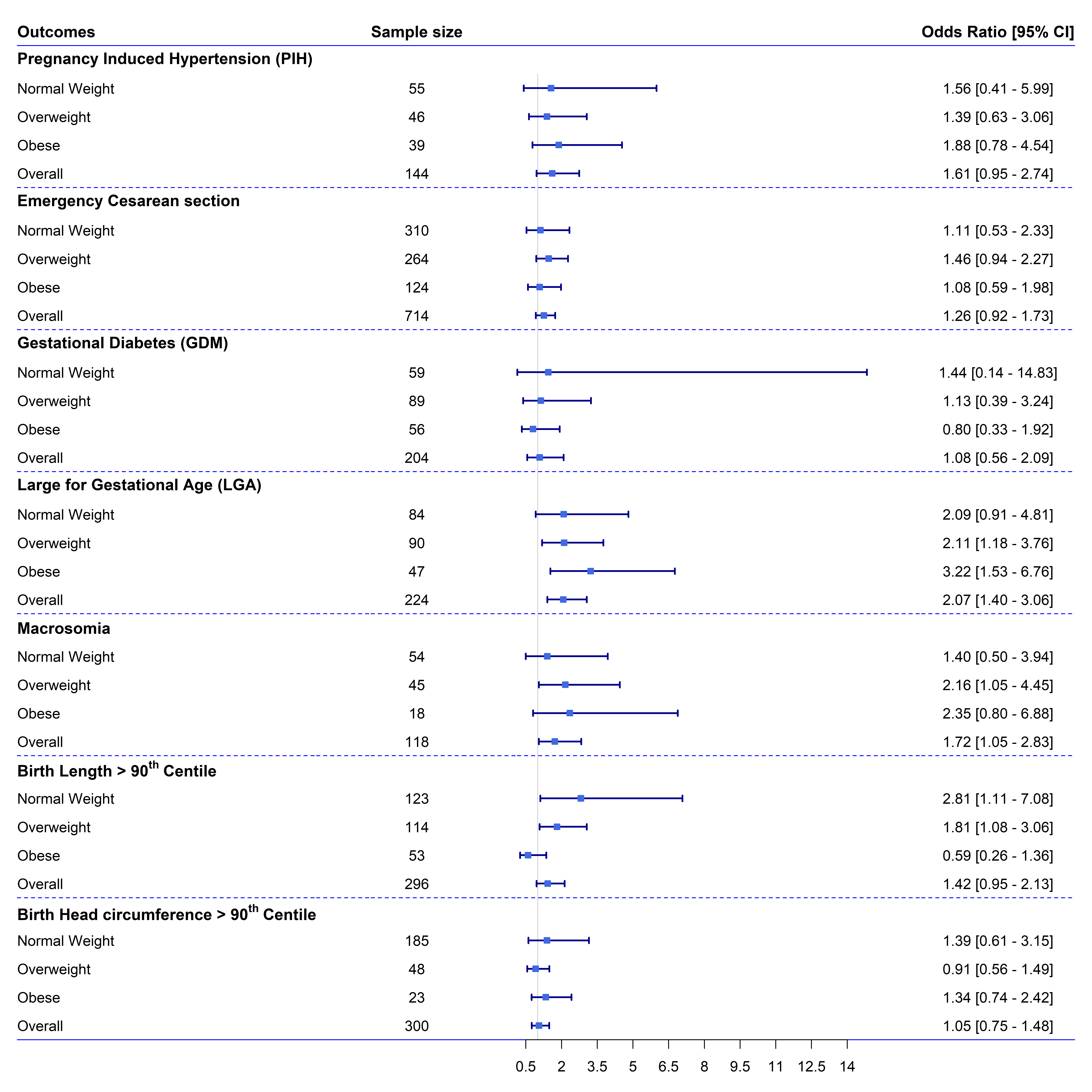


The odds ratios (ORs) and 95% confidence intervals (CIs) are shown for the association between gestational weight gain (GWG) above the maternal body mass index (BMI)-specific IOM standard during pregnancy (n=368) and adverse neonatal health outcomes. According to the IOM guidelines, GWG above the IOM standard is defined as follows: underweight women (<18.5 kg/m²) gaining more than 18 kg, normal weight women (18.5-24.9 kg/m²) gaining more than 16 kg, overweight women (25-29.9 kg/m²) gaining more than 11.5 kg, and women with obesity (≥30 kg/m²) gaining more than 9 kg during pregnancy. These associations were analyzed using multivariable regression models adjusted for possible covariates. The associations are shown across early-pregnancy BMI categories: normal weight (n=1686), overweight (n=1026), and obese (n=471). The association could not be shown for underweight women due to the small sample size. The prevalence of women gaining above the IOM standard was 55 for normal weight, 193 for overweight, and 119 for obese women. The reference group includes women gaining gestational weight within the IOM recommended range: underweight (12.5-18 kg), normal weight (11.5-16 kg), overweight (7-11.5 kg), and obese (5-9 kg) during pregnancy. Overall, 906 women gained IOM recommended weight (underweight, n=14, normal weight, n=322; overweight, n=392; and obese, n=178). For each outcome, the sample size represents the total number of women who experienced the outcome in each BMI category and in the overall sample.

# **Supplementary Figure S3:** Odds Ratio of gestational diabetes mellitus (GDM) with 95% confidence intervals (CI) for GWG centiles: <3^rd^, <5^th^, <10^th^, and <25^th^ compared to GWG between the 25^th^ and 75^th^ centiles from multivariable regression model adjusted for possible confounding factors: maternal age, maternal BMI, maternal education, smoking habit, and previous history of gestational diabetes.


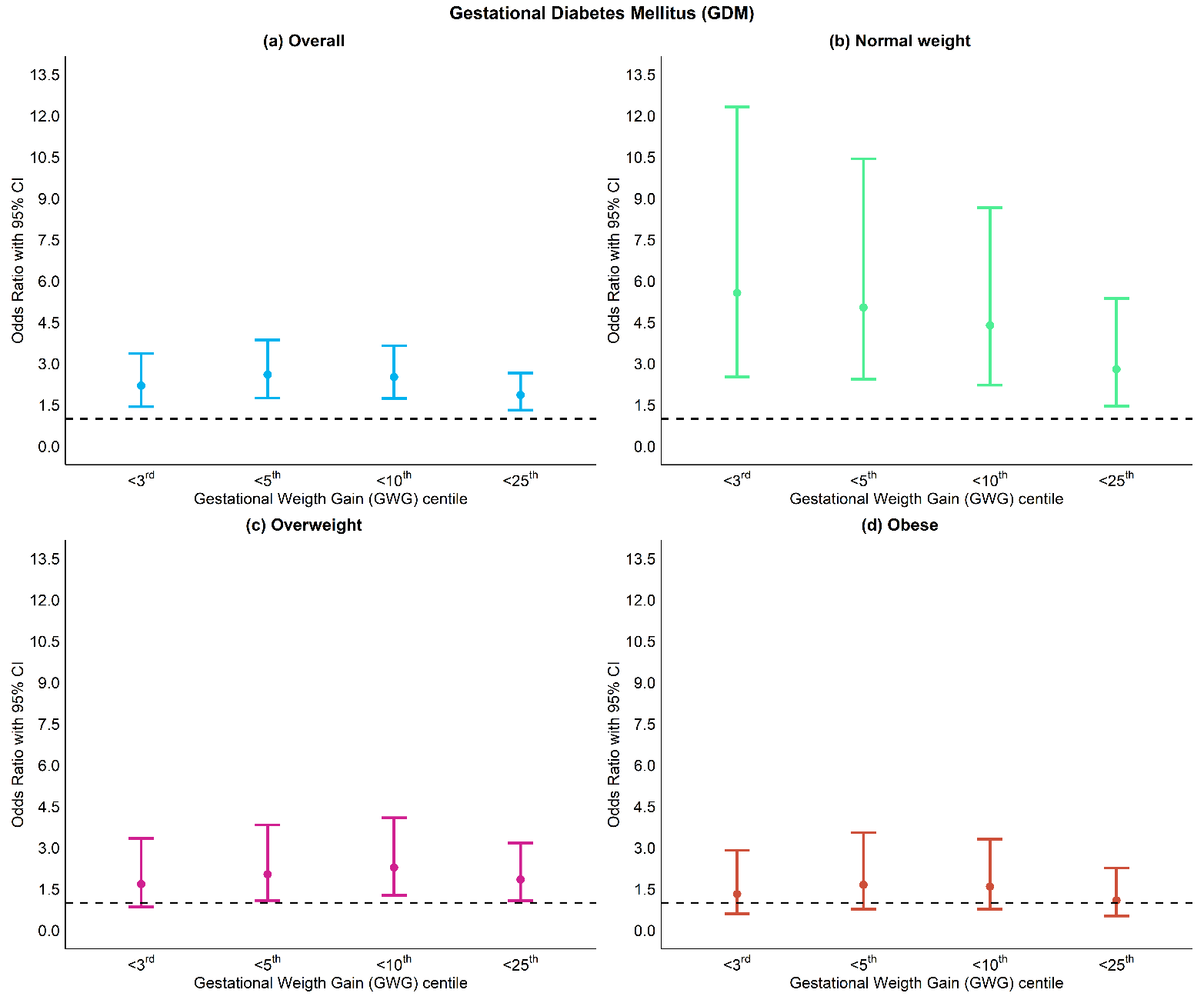


The odds ratios (ORs) and 95% confidence intervals (CIs) are shown for the association between continuous gestational weight gain (GWG) below the 3^rd^, 5^th^, 10^th^, and 25^th^ centiles of the gestational age-specific INTERGROWTH-21st (IG) standard throughout pregnancy and adverse perinatal health outcomes. These associations were analyzed using multivariable regression models adjusted for potential covariates. The results are presented across early-pregnancy BMI categories: normal weight (n = 1,686), overweight (n = 1,026), and obese (n = 471). The association could not be shown for underweight women due to the small sample size. The frequency of women gaining GWG below the 3^rd^ centile of the IG standard continuously throughout pregnancy was 367 for the overall sample, with 18 underweight, 125 normal weight, 558 overweight, and 312 obese women. For GWG below the 5^th^ centile, the frequency was 545 overall, with 27 underweight, 199 normal weight, 180 overweight, and 138 obese women. For GWG below the 10^th^ centile, the frequency was 926 overall, with 52 underweight, 373 normal weight, 302 overweight, and 198 obese women. For GWG below the 25^th^ centile, the frequency was 1,767 overall, with 94 underweight, 802 normal weight, 558 overweight, and 312 obese women. The reference group includes women who gained gestational weight between the 25^th^ and 75^th^ centiles of the IG standard during pregnancy (overall, n = 370; underweight, n = 20; normal weight, n = 216; overweight, n = 102; obese, n = 32). The prevalence of gestational diabetes mellitus (GDM) across the overall sample was n = 204; underweight, n = 0; normal weight, n = 59; overweight, n = 89; and obese, n = 56.

# **Supplementary Figure S4:** Odds Ratio of preterm birth, PTB (birth before 37 weeks of pregnancy) with 95% confidence intervals (CI) for GWG centiles: <3^rd^, <5^th^, <10^th^, and <25^th^ compared to GWG between the 25^th^ and 75^th^ centiles from multivariable regression model adjusted for potential confounders: maternal age, maternal BMI, maternal education, smoking habit, alcohol consumption, nulliparity, gestational diabetes, pregnancy induced hypertension, history of hypertension, diabetes, and infant sex.


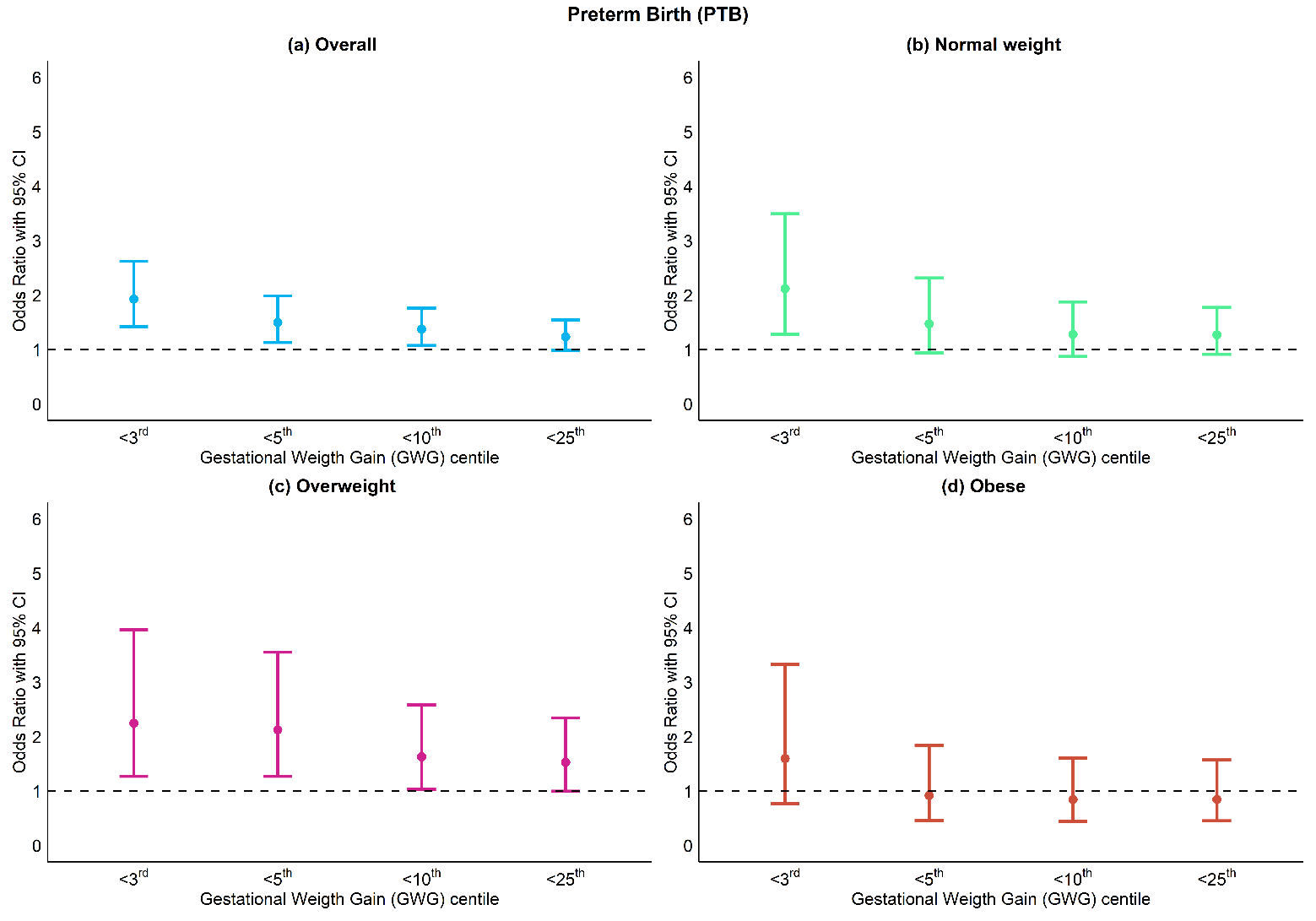


The odds ratios (ORs) and 95% confidence intervals (CIs) are shown for the association between continuous gestational weight gain (GWG) below the 3^rd^, 5^th^, 10^th^, and 25^th^ centiles of the gestational age-specific INTERGROWTH-21st (IG) standard throughout pregnancy and adverse perinatal health outcomes. These associations were analyzed using multivariable regression models adjusted for potential covariates. The results are presented across early-pregnancy BMI categories: normal weight (n = 1,686), overweight (n = 1,026), and obese (n = 471). The association could not be shown for underweight women due to the small sample size. The frequency of women gaining GWG below the 3^rd^ centile of the IG standard continuously throughout pregnancy was 367 for the overall sample, with 18 underweight, 125 normal weight, 558 overweight, and 312 obese women. For GWG below the 5^th^ centile, the frequency was 545 overall, with 27 underweight, 199 normal weight, 180 overweight, and 138 obese women. For GWG below the 10^th^ centile, the frequency was 926 overall, with 52 underweight, 373 normal weight, 302 overweight, and 198 obese women. For GWG below the 25^th^ centile, the frequency was 1,767 overall, with 94 underweight, 802 normal weight, 558 overweight, and 312 obese women. The reference group includes women who gained gestational weight between the 25^th^ and 75^th^ centiles of the IG standard during pregnancy (overall, n = 370; underweight, n = 20; normal weight, n = 216; overweight, n = 102; obese, n = 32). The prevalence of preterm birth (PTB) across the overall sample was n = 395; underweight, n = 24; normal weight, n = 163; overweight, n = 133; and obese, n = 75.

# **Supplementary Figure S5:** Odds Ratio of low birth weight, LBW (birthweight less than 2500g) with 95% confidence intervals (CI) for GWG centiles: <3^rd^, <5^th^, <10^th^, and <25^th^ compared to GWG between the 25^th^ and 75^th^ centiles from multivariable regression model adjusted for potential confounders: maternal age, maternal BMI, maternal education, smoking habit, alcohol consumption, nulliparity, gestational diabetes, pregnancy induced hypertension, history of hypertension, diabetes, and infant sex.


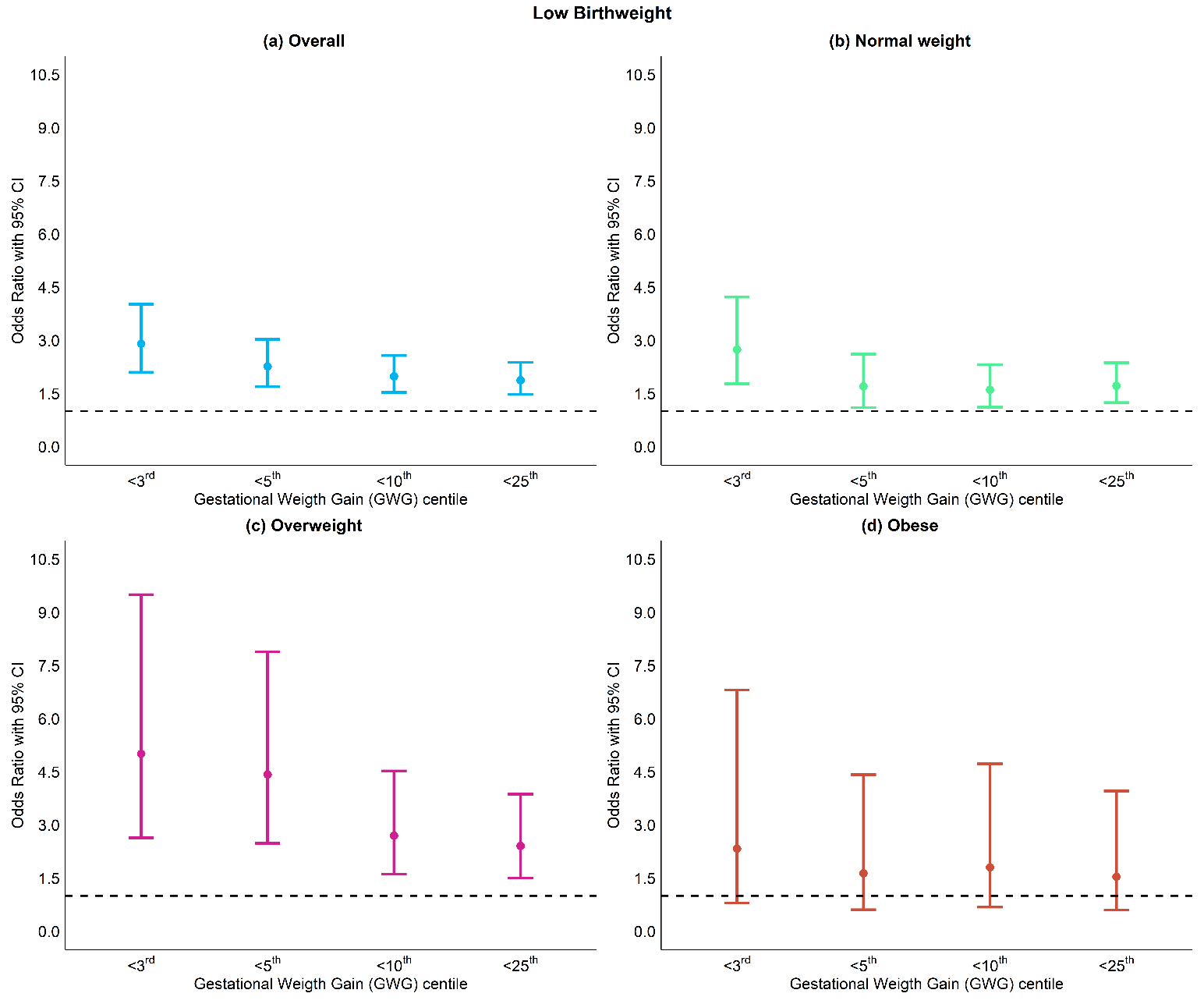


The odds ratios (ORs) and 95% confidence intervals (CIs) are shown for the association between continuous gestational weight gain (GWG) below the 3^rd^, 5^th^, 10^th^, and 25^th^ centiles of the gestational age-specific INTERGROWTH-21st (IG) standard throughout pregnancy and adverse perinatal health outcomes. These associations were analyzed using multivariable regression models adjusted for potential covariates. The results are presented across early-pregnancy BMI categories: normal weight (n = 1,686), overweight (n = 1,026), and obese (n = 471). The association could not be shown for underweight women due to the small sample size. The frequency of women gaining GWG below the 3^rd^ centile of the IG standard continuously throughout pregnancy was 367 for the overall sample, with 18 underweight, 125 normal weight, 558 overweight, and 312 obese women. For GWG below the 5^th^ centile, the frequency was 545 overall, with 27 underweight, 199 normal weight, 180 overweight, and 138 obese women. For GWG below the 10^th^ centile, the frequency was 926 overall, with 52 underweight, 373 normal weight, 302 overweight, and 198 obese women. For GWG below the 25^th^ centile, the frequency was 1,767 overall, with 94 underweight, 802 normal weight, 558 overweight, and 312 obese women. The reference group includes women who gained gestational weight between the 25^th^ and 75^th^ centiles of the IG standard during pregnancy (overall, n = 370; underweight, n = 20; normal weight, n = 216; overweight, n = 102; obese, n = 32). The prevalence of low birthweight (LBW) across the overall sample was n = 389; underweight, n = 39; normal weight, n = 185; overweight, n = 115; and obese, n = 50.

# **Supplementary Figure S6:** Odds Ratio of small for gestational age, SGA (birthweight below 10^th^ centile) with 95% confidence intervals (CI) for GWG centiles: <3^rd^, <5^th^, <10^th^, and <25^th^, compared to GWG between the 25^th^ and 75^th^ centiles from multivariable regression model adjusted for potential confounders: maternal age, maternal BMI, maternal education, smoking habit, alcohol consumption, nulliparity, gestational diabetes, pregnancy induced hypertension, history of hypertension, and infant sex.


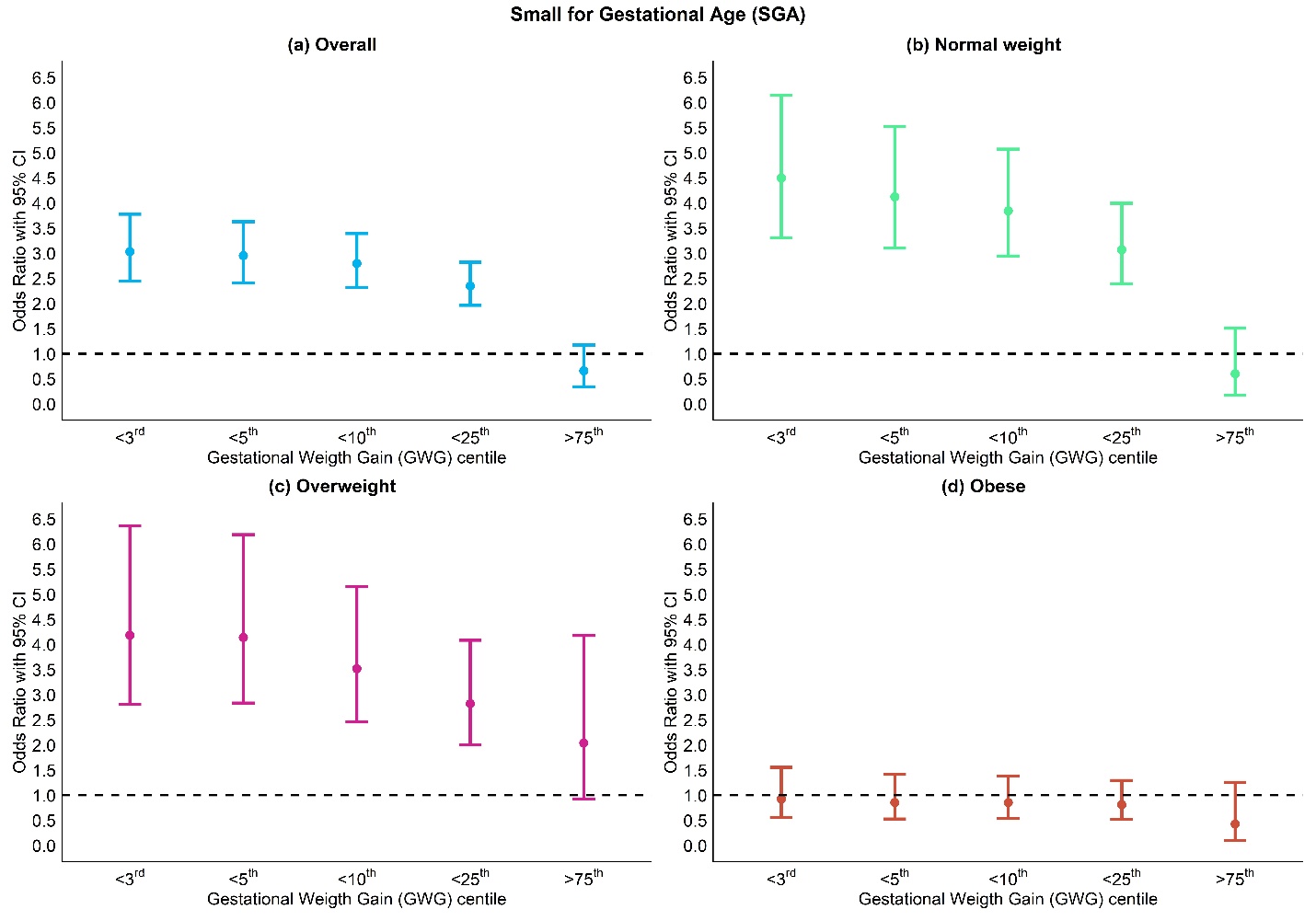


The odds ratios (ORs) and 95% confidence intervals (CIs) are shown for the association between continuous gestational weight gain (GWG) below the 3^rd^, 5^th^, 10^th^, and 25^th^ centiles of the gestational age-specific INTERGROWTH-21st (IG) standard throughout pregnancy and adverse perinatal health outcomes. These associations were analyzed using multivariable regression models adjusted for potential covariates. The results are presented across early-pregnancy BMI categories: normal weight (n = 1,686), overweight (n = 1,026), and obese (n = 471). The association could not be shown for underweight women due to the small sample size. The frequency of women gaining GWG below the 3^rd^ centile of the IG standard continuously throughout pregnancy was 367 for the overall sample, with 18 underweight, 125 normal weight, 558 overweight, and 312 obese women. For GWG below the 5^th^ centile, the frequency was 545 overall, with 27 underweight, 199 normal weight, 180 overweight, and 138 obese women. For GWG below the 10^th^ centile, the frequency was 926 overall, with 52 underweight, 373 normal weight, 302 overweight, and 198 obese women. For GWG below the 25^th^ centile, the frequency was 1,767 overall, with 94 underweight, 802 normal weight, 558 overweight, and 312 obese women. The reference group includes women who gained gestational weight between the 25^th^ and 75^th^ centiles of the IG standard during pregnancy (overall, n = 370; underweight, n = 20; normal weight, n = 216; overweight, n = 102; obese, n = 32). The prevalence of small for gestatoinal age (SGA) across the overall sample was n = 466; underweight, n = 43; normal weight, n = 255; overweight, n = 117; and obese, n = 51

# **Supplementary Figure S7:** Odds Ratio of birth length less than 10^th^ centile with 95% confidence intervals (CI) for GWG centiles: <3^rd^, <5^th^, <10^th^, and <25^th^ compared to GWG between the 25^th^ and 75^th^ centiles from multivariable regression model adjusted for potential confounders: maternal age, maternal BMI, maternal education, smoking habit, alcohol consumption, nulliparity, gestational diabetes, pregnancy induced hypertension, history of hypertension, diabetes, and infant sex.


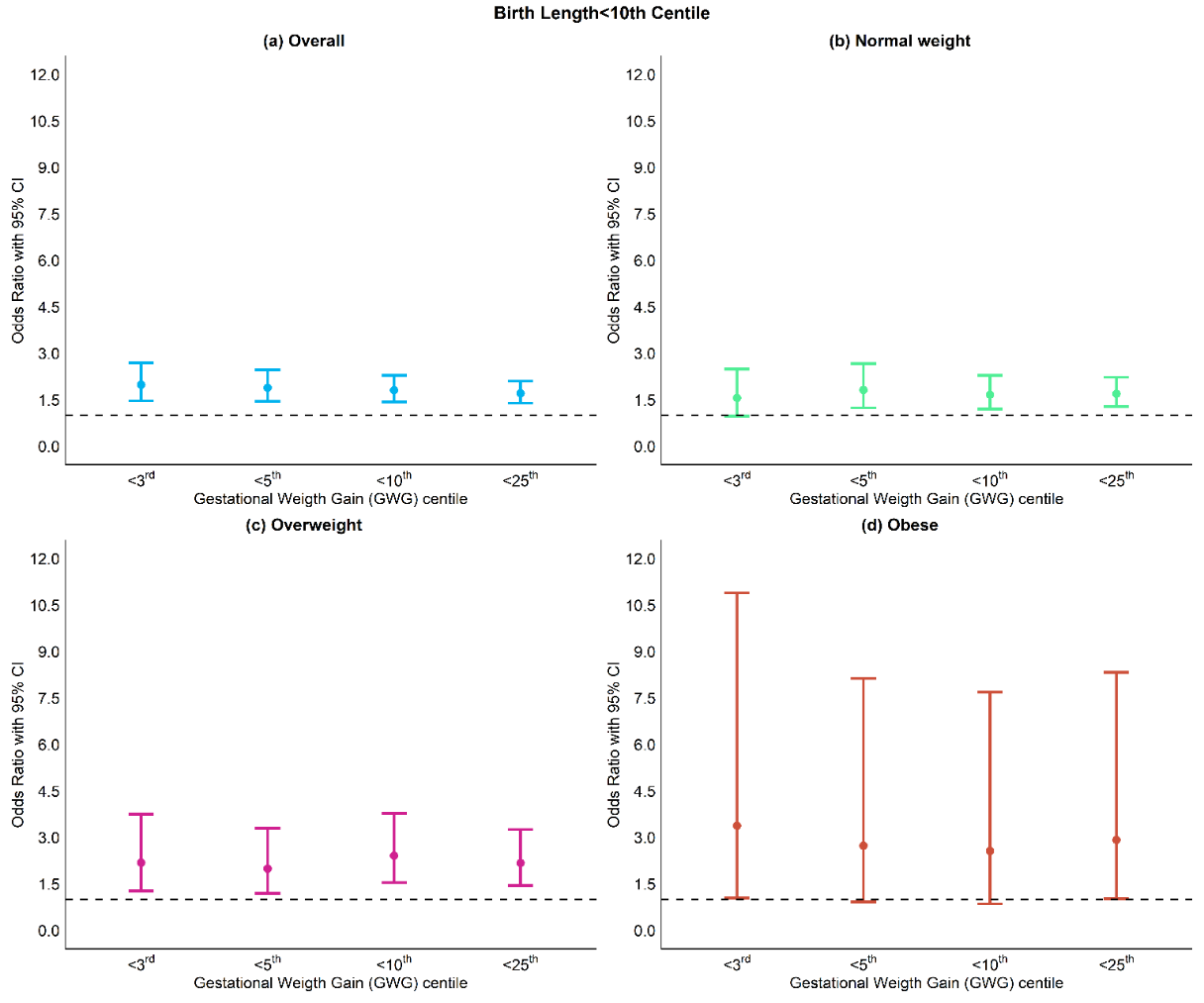


The odds ratios (ORs) and 95% confidence intervals (CIs) are shown for the association between continuous gestational weight gain (GWG) below the 3^rd^, 5^th^, 10^th^, and 25^th^ centiles of the gestational age-specific INTERGROWTH-21st (IG) standard throughout pregnancy and adverse perinatal health outcomes. These associations were analyzed using multivariable regression models adjusted for potential covariates. The results are presented across early-pregnancy BMI categories: normal weight (n = 1,686), overweight (n = 1,026), and obese (n = 471). The association could not be shown for underweight women due to the small sample size. The frequency of women gaining GWG below the 3^rd^ centile of the IG standard continuously throughout pregnancy was 367 for the overall sample, with 18 underweight, 125 normal weight, 558 overweight, and 312 obese women. For GWG below the 5^th^ centile, the frequency was 545 overall, with 27 underweight, 199 normal weight, 180 overweight, and 138 obese women. For GWG below the 10^th^ centile, the frequency was 926 overall, with 52 underweight, 373 normal weight, 302 overweight, and 198 obese women. For GWG below the 25^th^ centile, the frequency was 1,767 overall, with 94 underweight, 802 normal weight, 558 overweight, and 312 obese women. The reference group includes women who gained gestational weight between the 25^th^ and 75^th^ centiles of the IG standard during pregnancy (overall, n = 370; underweight, n = 20; normal weight, n = 216; overweight, n = 102; obese, n = 32). The prevalence of birth length <10^th^ cnetile across the overall sample was n = 432; underweight, n = 36; normal weight, n = 232; overweight, n = 120; and obese, n = 44.

# **Supplementary Figure S8:** Odds Ratio of birth head circumference less than 10^th^ centile with 95% confidence intervals (CI) for GWG centiles: <3^rd^, <5^th^, <10^th^, and <25^th^ compared to GWG between the 25^th^ and 75^th^ centiles from multivariable regression model adjusted for potential confounders: maternal age, maternal BMI, maternal education, smoking habit, alcohol consumption, nulliparity, gestational diabetes, pregnancy induced hypertension, history of hypertension, diabetes, and infant sex.


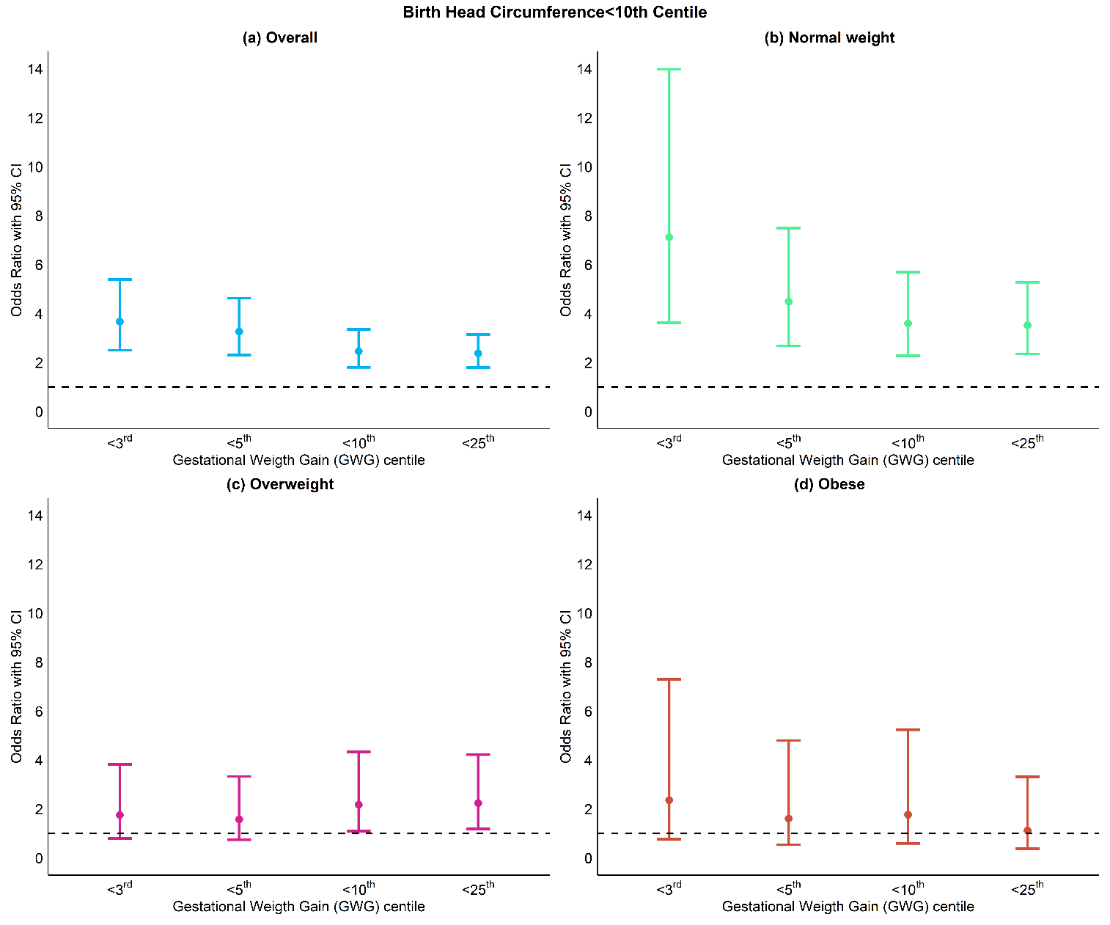


The odds ratios (ORs) and 95% confidence intervals (CIs) are shown for the association between continuous gestational weight gain (GWG) below the 3^rd^, 5^th^, 10^th^, and 25^th^ centiles of the gestational age-specific INTERGROWTH-21st (IG) standard throughout pregnancy and adverse perinatal health outcomes. These associations were analyzed using multivariable regression models adjusted for potential covariates. The results are presented across early-pregnancy BMI categories: normal weight (n = 1,686), overweight (n = 1,026), and obese (n = 471). The association could not be shown for underweight women due to the small sample size. The frequency of women gaining GWG below the 3^rd^ centile of the IG standard continuously throughout pregnancy was 367 for the overall sample, with 18 underweight, 125 normal weight, 558 overweight, and 312 obese women. For GWG below the 5^th^ centile, the frequency was 545 overall, with 27 underweight, 199 normal weight, 180 overweight, and 138 obese women. For GWG below the 10^th^ centile, the frequency was 926 overall, with 52 underweight, 373 normal weight, 302 overweight, and 198 obese women. For GWG below the 25^th^ centile, the frequency was 1,767 overall, with 94 underweight, 802 normal weight, 558 overweight, and 312 obese women. The reference group includes women who gained gestational weight between the 25^th^ and 75^th^ centiles of the IG standard during pregnancy (overall, n = 370; underweight, n = 20; normal weight, n = 216; overweight, n = 102; obese, n = 32). The prevalence of birth head circumference <10^th^ cnetile across the overall sample was n = 300; underweight, n = 44; normal weight, n = 185; overweight, n = 48; and obese, n = 23.

# **Supplementary Table S5**: Maternal and neonatal health outcomes by gestational weight gain categories in a cohort of 3354 women

|  | | **Consistently below or greater than the threshold throughout the pregnancy** | | **IG standards at any point of pregnancy** | | **IOM recommendations*** | |
| --- | --- | --- | --- | --- | --- | --- | --- |
| **Perinatal Health Outcomes** | **Overall,  n = 3,354** | **GWG<25^th^ centile,**  **n = 1,767** | **GWG>75^th^ centile,**  **n = 23** | **GWG<25^th^ centile,**  **n = 2,801** | **GWG>75^th^ centile,**  **n = 243** | **GWG<IOM,**  **n = 2,079** | **GWG> IOM,**  **n = 368** |
| **Maternal health outcomes** |  |  |  |  |  |  |  |
| Pregnancy induced hypertension | 144 (4.3%) | 62 (3.5%) | 3 (13.0%) | 111 (4.0%) | 23 (9.5%) | 76 (3.7%) | 30 (8.2%) |
| Gestation diabetes | 204 (6.1%) | 142 (8.0%) | 0 (0%) | 184 (6.6%) | 12 (4.9%) | 147 (7.1%) | 16 (4.3%) |
| Emergency C-section | 714 (21.3%) | 349 (19.8%) | 9 (39.1%) | 578 (20.6%) | 67 (27.6%) | 399 (19.2%) | 99 (26.9%) |
| **Neonatal health outcomes** |  |  |  |  |  |  |  |
| Preterm birth | 395 (11.8%) | 246 (13.9%) | 4 (17.4%) | 339 (12.1%) | 17 (7.0%) | 312 (15.0%) | 13 (3.5%) |
| Low birthweight | 389 (11.6%) | 257 (14.5%) | 3 (13.0%) | 346 (12.4%) | 14 (5.8%) | 318 (15.3%) | 12 (3.3%) |
| Macrosomia | 118 (3.5%) | 32 (1.8%) | 0 (0%) | 83 (3.0%) | 18 (7.4%) | 38 (1.8%) | 34 (9.2%) |
| Small for gestational age | 466 (13.9%) | 300 (17.0%) | 4 (17.4%) | 417 (14.9%) | 19 (7.8%) | 351 (16.9%) | 31 (8.4%) |
| Large for gestational age | 224 (6.7%) | 75 (4.2%) | 0 (0%) | 156 (5.6%) | 32 (13.2%) | 89 (4.3%) | 62 (16.8%) |
| Birth length<10^th^ centile | 432 (12.9%) | 256 (14.5%) | 2 (8.7%) | 382 (13.6%) | 22 (9.1%) | 311 (15.0%) | 33 (9.0%) |
| Birth length >90^th^ centile | 296 (8.8%) | 146 (8.3%) | 0 (0%) | 242 (8.6%) | 21 (8.6%) | 163 (7.8%) | 48 (13.0%) |
| Birth head circumference<10^th^ centile | 300 (8.9%) | 189 (10.7%) | 1 (4.3%) | 275 (9.8%) | 7 (2.9%) | 247 (11.9%) | 12 (3.3%) |
| Birth head circumference> 90^th^ centile | 410 (12.2%) | 181 (10.2%) | 3 (13.0%) | 327 (11.7%) | 43 (17.7%) | 192 (9.2%) | 70 (19.0%) |

IG standards are gestational age specific. IOM recommended range: underweight (12.5-18 kg), normal weight (11.5-16 kg), overweight (7-11.5 kg), and obese (5-9 kg) during pregnancy. According to the IOM guidelines, GWG below the IOM standard is defined as follows: underweight women (<18.5 kg/m²) gaining less than 12.5 kg, normal weight women (18.5-24.9 kg/m²) gaining less than 11.5 kg, overweight women (25-29.9 kg/m²) gaining less than 7 kg, and women with obesity (≥30 kg/m²) gaining less than 5 kg during pregnancy. According to the IOM guidelines, GWG above the IOM standard is defined as follows: underweight women (<18.5 kg/m²) gaining more than 18 kg, normal weight women (18.5-24.9 kg/m²) gaining more than 16 kg, overweight women (25-29.9 kg/m²) gaining more than 11.5 kg, and women with obesity (≥30 kg/m²) gaining more than 9 kg during pregnancy.

# **Supplementary Figure S9**: Adjusted odds ratios (ORs) with 95% confidence interval (CIs) of maternal and neonatal health outcomes for women gaining consistently <25^th^ centile GWG throughout pregnancy. These odds ratios were performed in the overall sample and in high- (UK) and low-income countries (Brazil, Kenya, Pakistan, South Africa and Thailand).


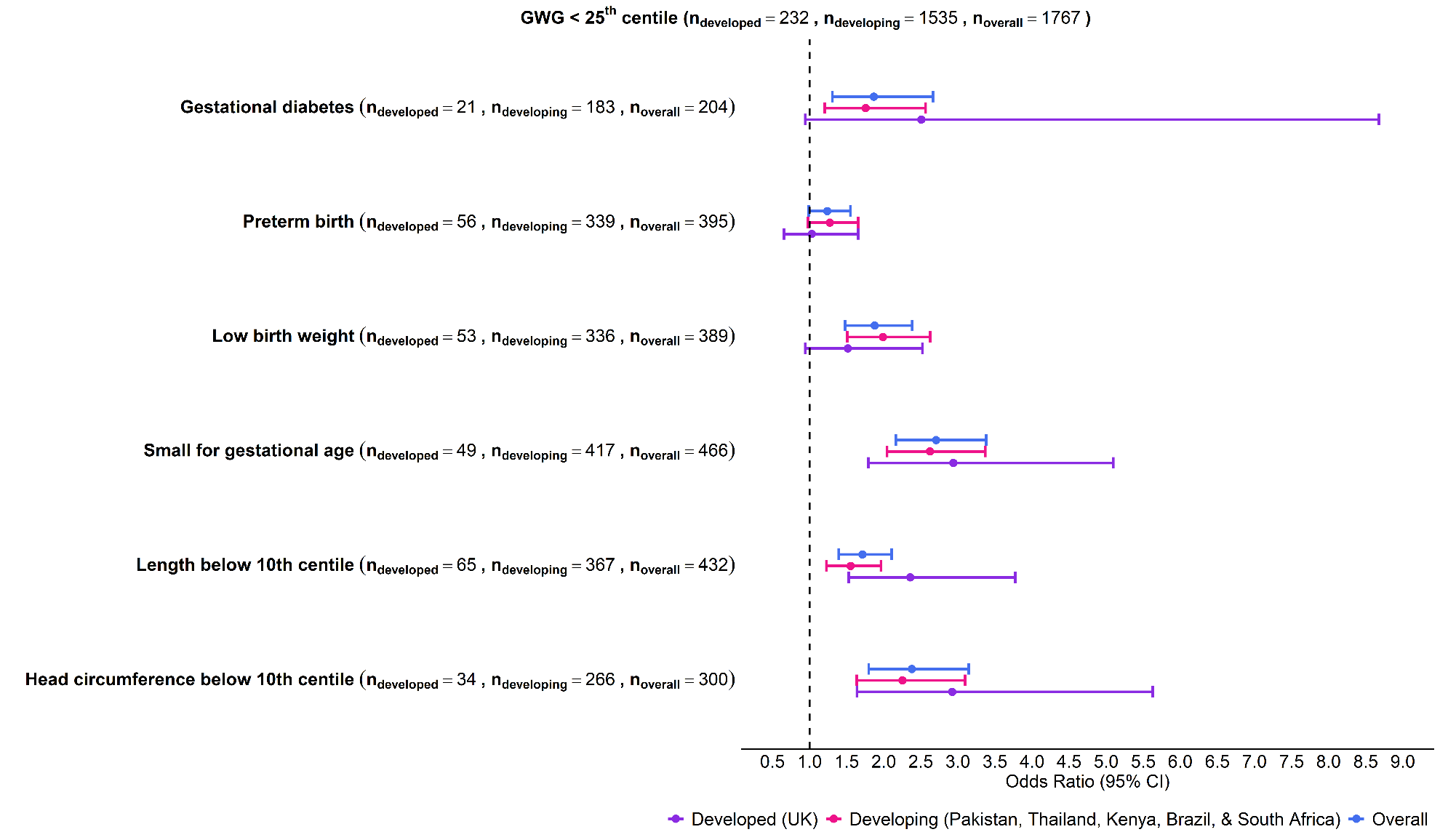


# **Supplementary Figure S10**: Adjusted odds ratio with 95% confidence interval of maternal and neonatal health outcomes for women gaining consistently <25^th^ centile GWG throughout the pregnancy. The odds ratio were performed excluding each site at a time.

***
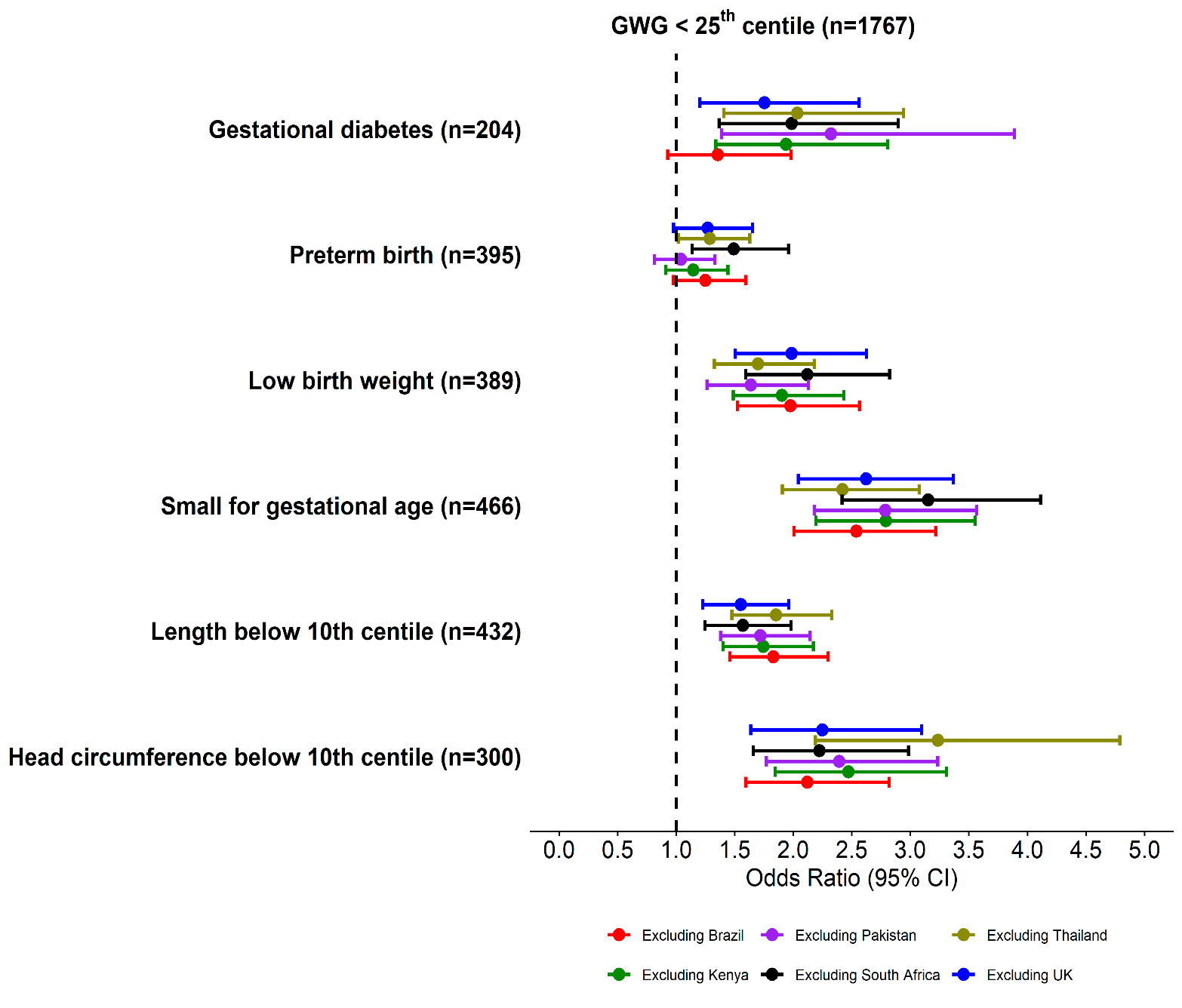
***

# **Supplementary Figure S11**: Adjusted odds ratio with 95% confidence interval of maternal and neonatal health outcomes for women gaining consistently <25^th^ centile GWG throughout the pregnancy. The odds ratio were performed in the overall sample and in study sites that contributed to INTERGROWTH-21^st^ (IG) study (UK, Kenya, and Brazil) and sites that contributed only to the INTERBIO-21^st^ study (Pakistan, South Africa, and Thailand).


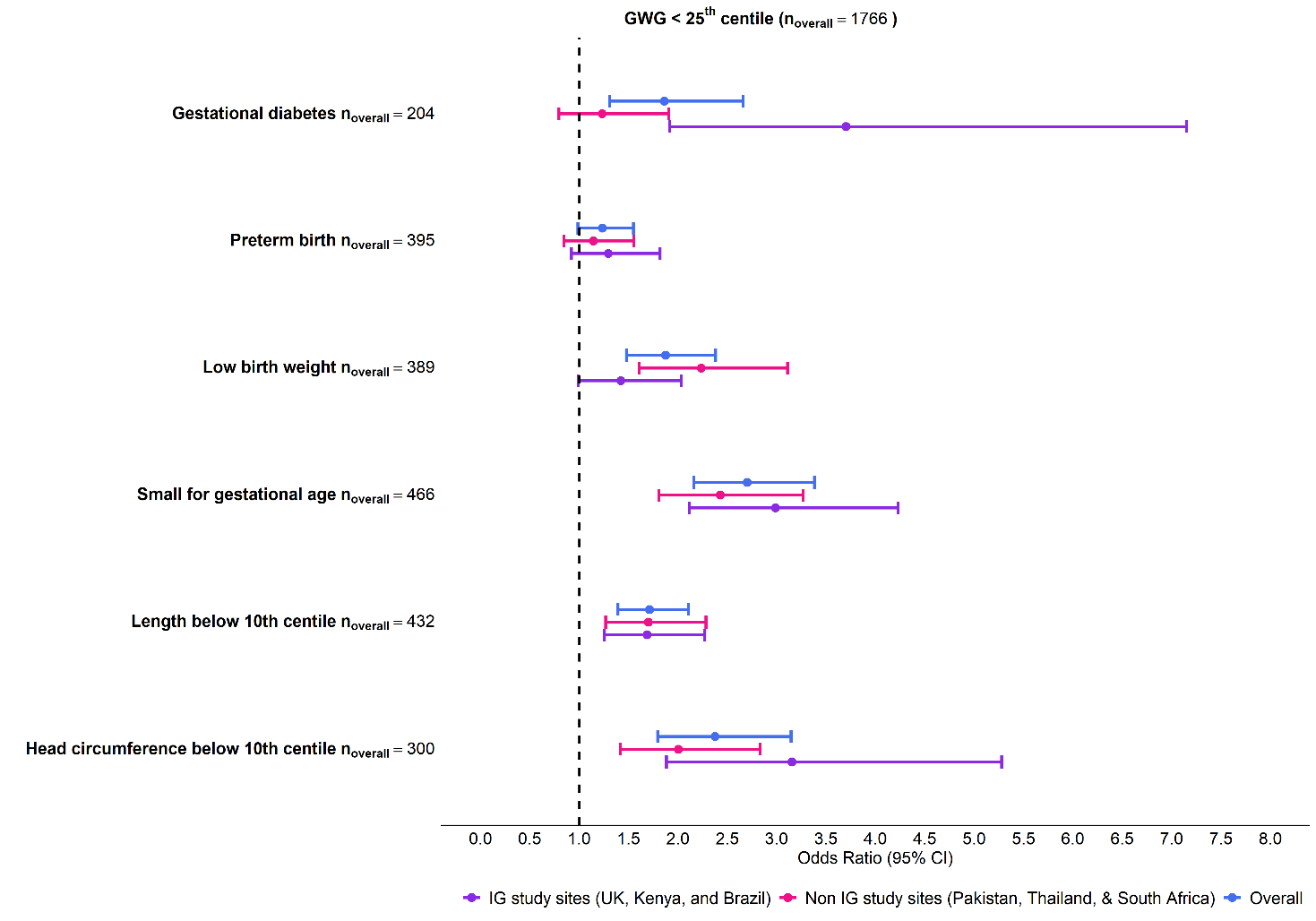


This plot shows the risk of adverse health outcomes in the overall sample of INTERBIO-21^st^ Fetal study that contributed to this analysis. Of the six study sites included, three countries (UK, Kenya, and Brazil) also contributed to the INTERGROWTH-21^st^ (IG) study, while the other three countries (Pakistan, Thailand, South Africa) did not. The GWG reference used in this study was derived from IG study. As a sensitivity analysis, we examined the association between GWG and adverse health outcomes both in IG study sites and non-IG study sites. Prevalence of the outcomes by study sites is as follows: gestational diabetes Mellitus, GDM (n_IG sites_=63, n_non IG sites_=141); preterm birth, PTB (n_IG sites_=139, n_non IG sites_=256); low birth weight, LBW (n_IG sites_=120, n_non IG sites_=269); small for gestational age, SGA (n_IG sites_=148, n_non IG sites_=318); birth length <10^th^ centile (n_IG sites_=151, n_non IG sites_=281); and birth head circumference <10^th^ centile (n_IG sites_=69, n_non IG sites_=231).

# **Supplementary Table S6**: Adjusted odds ration with 95% confidence interval (CI) of maternal and neonatal health outcomes for gestational weight gain centiles by trimester for 3354 women

|  | **Second Trimester, n=3228** | **Third Trimester, n=3254** |
| --- | --- | --- |
|  | **GWG<25th centile,**  **N =** **2221** | **GWG<25th centile,**  **N= 1860** |
| *Predictors* | *Odds Ratios (95% CI)* | *Odds Ratios (95% CI)* |
| Gestational diabetes, n=204 | 1.51 | 1.71 |
|  | (0.81 – 2.83) | (0.89 – 3.29) |
| Preterm birth, n=395 | 1.00 | 1.43 |
|  | (0.70 – 1.44) | (0.93 – 2.19) |
| Low birthweight, n=389 | 1.45 | 1.96 |
|  | (0.97 – 2.16) | (1.24 – 3.08) |
| Small for gestational age, n=466 | 2.12 | 2.54 |
|  | (1.39 – 3.23) | (1.63 – 3.93) |
| Birth length <10th centile, n=432 | 1.50 | 1.66 |
|  | (1.01 – 2.23) | (1.11 – 2.49) |
| Birth head circumference <10th centile, n=300 | 2.18 | 2.40 |
|  | (1.25 – 3.80) | (1.37 – 4.21) |
|  | **Second Trimester, n=3228** | **Third Trimester, n=3254** |
|  | **GWG>75th centile,**  **N=34** | **GWG >75th centile,**  **N=87** |
|  | *Odds Ratios (95% CI)* | *Odds Ratios (95% CI)* |
|  |  |  |
| Pregnancy induced hypertension, n=144 | 2.20 | 2.52 |
|  | (0.55 – 8.86) | (1.07 – 5.96) |
| Gestational diabetes, n=204 | 0.44  (0.04 – 4.39) | 0.74  (0.14 – 3.84) |
| Emergency cesarean delivery, n=714 | 1.28  (0.53 – 3.14) | 1.84  (1.02 – 3.39) |
|  |  |  |
| Large for gestational age, n=224 | 0.69 | 1.43 |
|  | (0.19 – 2.47) | (0.71 – 2.88) |
| Macrosomia, n=118 | 0.56 | 1.24 |
|  | (0.07 – 4.60) | (0.48 – 3.23) |
| Birth length >90th centile, n=410 | **** | 1.37 |
|  |  | (0.62 – 3.03) |
| Birth head circumference >90th centile, n=410 | 0.45 | 1.12 |
|  | (0.12 – 1.69) | (0.57 – 2.21) |

The odds ratios (ORs) and 95% confidence intervals (CIs) are shown for the association between gestational weight gain (GWG) continuously below the 25^th^ or above 75^th^ centile of the gestational age-specific INTERGROWTH-21st (IG) standard during second trimester and third trimester of pregnancy and adverse perinatal health outcomes. These associations were analyzed using multivariable regression models adjusted for possible covariates. During second trimester 2221women and in third trimester 1860 women gained consistently <25^th^ centile GWG IG standard. Whereas 37 women in second trimester and 87 women in third trimester gained more than 75^th^ centile GWG of IG standard. The reference group includes women gaining gestational weight between the 25th and 75th centiles of the IG standard during second trimester (n=948) and third trimester (n=691).
